# Promoting Psychological Resilience and Well-Being in Youth With a Smartphone-Based Ecological Momentary mHealth Intervention: Secondary Analysis of a Microrandomized Trial

**DOI:** 10.2196/85552

**Published:** 2026-06-18

**Authors:** Eva Wierzba, Anita Schick, Christian Rauschenberg, Janik Fechtelpeter, Selina Hiller, Christian Götzl, Daniel Durstewitz, Silvia Krumm, Georgia Koppe, Ulrich Reininghaus

**Affiliations:** 1 Department of Public Mental Health, Central Institute of Mental Health, Medical Faculty Mannheim, Heidelberg University, J5, Mannheim, 68159, Germany, 49 6211703 ext 1930; 2 German Center for Mental Health (DZPG), Partner Site Mannheim-Heidelberg-Ulm, Mannheim, Germany; 3 Department of Theoretical Neuroscience, Central Institute of Mental Health, Medical Faculty Mannheim, Heidelberg University, Mannheim, Germany; 4 Hector Institute for Artificial Intelligence in Psychiatry, Central Institute of Mental Health, Medical Faculty Mannheim, Heidelberg University, Heidelberg, Germany; 5 Department of Psychiatry and Psychotherapy, Central Institute of Mental Health, Medical Faculty Mannheim, Heidelberg University, Mannheim, Germany; 6 RG (Research Group) Machine Learning Human Behavior, Interdisciplinary Center for Scientific Computing, Heidelberg University, Heidelberg, Germany; 7 Department of Psychiatry and Psychotherapy, School of Medicine and Health, Technical University of Munich, TUM University Hospital, Munich, Germany; 8 German Center for Mental Health (DZPG), Partner Site Munich-Augsburg, Munich, Germany; 9 Department of Forensic Psychiatry and Psychotherapy, University of Ulm and BKH Guenzburg, Ulm, Germany; 10 Interdisciplinary Center for Scientific Computing, Heidelberg University, Heidelberg, Germany; 11 Department of Psychiatry and Psychotherapy, Medical Faculty, Leipzig University, Leipzig, Germany; 12 Hertie Institute for AI (Artificial Intelligence) in Brain Health, University of Tübingen, Tübingen, Germany; 13 Health Service and Population Research Department, Institute of Psychiatry, Psychology & Neuroscience, King's College London, London, United Kingdom; 14 ESRC (Economic and Social Research Council) Centre for Society and Mental Health, King's College London, London, United Kingdom

**Keywords:** ecological momentary assessment, experience sampling method, ambulatory assessment, ecological momentary intervention, just-in-time adaptive intervention, digital training, mobile health, digital health, mental health, affect, adolescent, mobile phone

## Abstract

**Background:**

Ecological momentary interventions (EMIs) may be a promising tool for promoting mental well-being in youth, as they allow for targeting resilience and other protective factors in daily life.

**Objective:**

In this secondary analysis of a microrandomized trial, we explored proximal effects of a coach-guided compassion-focused smartphone-based digital training for young people applying principles and techniques of EMI (the AI4U [artificial intelligence for personalized digital mental health promotion in youth] training) on momentary outcomes of mental well-being.

**Methods:**

A convenience sample of participants aged 14 to 25 years was recruited mostly via an open-access website and completed up to 6 self-report ecological momentary assessments (EMA) per day to measure their momentary mental well-being (ie, positive affect, negative affect, and stress) during the 30-day AI4U training phase. Some EMA prompts triggered EMI components to promote their momentary resilience and well-being. Multilevel modeling was used to analyze proximal effects of initiating EMI components on outcomes assessed at the next time point, the potential moderating effect of momentary affect and stress at the time of EMI initiation, and the potential mediating effect of change in momentary resilience on the effect of EMI initiation on changes in momentary affect and stress.

**Results:**

A total of 170 individuals completed 13,059 EMA prompts and initiated 6667 EMI components. No evidence was found that momentary outcomes of mental well-being at a time point differed depending on whether an EMI component was initiated at the previous time point vs when no EMI component was initiated at the previous time point (positive affect: b=0.00, 95% CI −0.04 to 0.04; negative affect: b=−0.03, 95% CI −0.06 to 0.01; stress: b=0.01, 95% CI −0.03 to 0.05). There was no strong difference in the magnitude of this effect when momentary mental well-being at the time of EMI initiation was high vs low (positive affect: b=0.08, 95% CI 0.01 to 0.16; negative affect: b=−0.09, 95% CI −0.17 to −0.01; stress: b=0.09, 95% CI 0.01 to 0.17). Changes in momentary resilience did not mediate the effect of EMI component initiation (vs EMI component noninitiation) on changes in momentary mental well-being (positive affect: b=0.00, 95% CI −0.01 to 0.02; negative affect: b=−0.00, 95% CI −0.01 to 0.01; stress: b=0.00; 95% CI 0.00 to 0.00).

**Conclusions:**

By investigating proximal effects of EMI components, this secondary analysis analyzes proximal effects of a novel digital training using EMA, which presents an innovative approach to understanding how the digital training leads to long-term improvement of distal outcomes. The analysis contributes to research in the field of EMI by serving as a basis for future investigations on the momentary effects of EMI components, which can support the development of scalable interventions to promote mental well-being in the public.

## Introduction

### Background and Rationale

Young individuals are a vulnerable group at risk for mental health conditions, as many mental health conditions first appear in childhood or adolescence [1,2] and contribute significantly to the disease burden of young people [3]. In the past years, young individuals have also been facing a multitude of crises, such as the COVID-19 pandemic and the climate crisis, which are associated with negative mental health outcomes [4]. For instance, a systematic review showed that the prevalence of depressive and anxiety symptoms among children and adolescents increased during the COVID-19 pandemic compared to prepandemic numbers [5]. Hence, there is an urgent need for easily accessible measures of mental health promotion in youth.

A promising approach involves targeting psychological resilience, as numerous studies have demonstrated a strong association between higher levels of resilience and improved mental health and well-being [6,7]. Psychological resilience can be defined as the ability to deal with adversity in different areas of life while maintaining focus on one’s personal goals [8,9]. It is therefore an especially important capability to protect mental health in times of crisis. Different approaches exist to increase resilience, such as interventions based on cognitive behavioral therapy (CBT) or mindfulness [10]. However, these interventions are often associated with high costs and time investment [10]. In addition, young people can be hard to reach for interventions aimed at improving their mental well-being due to, for example, stigma or low mental health literacy [11].

Digital interventions have the potential to increase the accessibility of mental health services for young people [12] and to reduce costs [13]. A review showed that digital resilience programs have positive effects on, for example, psychological distress and positive mental health in nonclinical adult populations [14]. A specific type of digital intervention are ecological momentary interventions (EMIs), which deliver intervention components in real time via digital platforms and apps and can therefore be directly incorporated in everyday life [15-17]. They can be tailored to the specific needs of an individual and to the requirements of a given context [18,19] based on intensive longitudinal data collected using ecological momentary assessment (EMA) [20-23].

The AI4U (artificial intelligence for personalized digital mental health promotion in youth) training, a digital mHealth (mobile health) training that was developed as part of the living lab AI4U applies principles of both EMA and EMI. The content and design of the EMI components were informed by EMIs implemented in previous studies, that is, SELFIE [21,24], EMIcompass [20,25], and PerPAIN [26]. The EMI components consist of short exercises that are based on positive refocusing, positive imagery, mindfulness, and compassion-focused techniques, such as breathing exercises and a journal of joyful moments (for a full description of the EMI components, see Multimedia Appendix 1). The components aim to improve resilience, emotion regulation, and a compassionate self-image, and thereby to promote mental health and well-being. Findings from the primary analysis of the living lab AI4U suggest beneficial effects of the AI4U training on distal indicators of mental health and well-being at post intervention (compared to baseline), that is, reduced psychological distress, improved resilience, and increased use of adaptive emotion regulation strategies [27].

EMIs have been found to be effective in the promotion of distal indicators of mental health in other research: a review found that EMI based on CBT can improve mental well-being and reduce symptoms of mental health conditions in both healthy and clinical samples [28]. Another review suggested that EMIs targeting outcomes of mental health and positive psychological well-being have small to medium-sized effects, but that the low quality of the available studies requires more rigorous evidence generated by randomized controlled trials (RCTs) [29]. A recent RCT has shown that a mindfulness-based EMI led to a reduction in anxiety and depressive symptoms in individuals with generalized anxiety disorder after 2 weeks of training [30]. In another RCT, participants’ self-esteem after using the SELFIE intervention for 6 weeks was, on average, higher both at postintervention and at 6-month follow-up compared to the control condition that received care as usual [21]. An EMI with compassion-focused exercises improved quality of life and stress-reactivity after 6 weeks of training in a feasibility RCT [20].

Despite the evidence on the effects of EMIs for improving outcomes of mental health and psychological well-being, so far, it is unclear how exactly EMIs yield these beneficial effects. Understanding the exact mechanisms through which EMIs work would offer the opportunity to further adapt EMIs to increase their efficacy. One way to elucidate this further is to shed light on the immediate effects of individual EMI components, often referred to as proximal effects [31]. Proximal effects of EMIs were reported in previous studies: an EMI targeted to reduce smoking had a proximal effect on negative affect [32]. Furthermore, the results of a process evaluation of the EMIcompass intervention indicated that strengthening the soothing system, which may reflect resilience [33], may have accounted for the initial signals of reduced stress reactivity and improved quality of life that were observed in the feasibility RCT [20,33]. In a subsample of participants with early mental health problems, initial signals for proximal effects of the EMIcompass intervention on negative affect, stress, and stress reactivity were found [34].

In the AI4U training, EMI components that aim at improving resilience are easily integrated into daily life to facilitate the ecological translation of the strategies learned. Building on previous research [20,32,33], completing EMI components in the moment is intended to yield a proximal effect on momentary mental well-being, such as affect and stress. Specifically, executing strategies that aim at improving resilience may help individuals to mitigate their negative affect, to amplify their positive affect, and to reduce their perceived stress [6,7,34]. As resilience can be defined as the ability to deal with and overcome adversity [8,9], applying EMI components in the moment may be posited to be beneficial in adverse situations when individuals need to cope with negative feelings or stress.

### Objectives

The aim of this secondary analysis of data collected as part of the AI4U living lab was to investigate proximal effects of initiating EMI components, that is, effects of EMI components on outcomes assessed at the next time point using EMA in youths. It was expected that initiating an EMI component, which means applying strategies aimed toward improving resilience, would improve momentary indicators of mental well-being, that is, positive affect, negative affect, and stress at the subsequent EMA time point. We assume that initiating an EMI component aimed at improving resilience is especially useful in situations that require the individual to deal with adversity. Therefore, the effect of the initiation of an EMI component was expected to be greater when mental well-being at the time of initiating the EMI was low compared to when it was high. In addition, it was assumed that the effect of initiating EMI components on mental well-being would be partially mediated by an increase in momentary resilience following the initiation of the EMI. Therefore, three hypotheses were tested.

First, it was hypothesized that (1) positive affect at t_n_ will be higher following the initiation of an EMI component at t_n–1_ compared with noninitiation of an EMI component at t_n–1_, (2) negative affect, and (3) perceived stress at t_n_ will be lower following initiation of an EMI component at t_n–1_ compared with noninitiation of an EMI component at t_n–1_.

Second, it was hypothesized that the magnitude of this difference in (1) positive affect, (2) negative affect, and (3) perceived stress between initiation and noninitiation of EMI components at t_n–1_ will be modified by levels of (1) positive affect, (2) negative affect, and (3) perceived stress at t_n–1_ such that the magnitude of this difference will be greater following (1) low levels of positive affect, (2) high levels of negative affect, and (3) high levels of perceived stress at t_n–1_ compared with (1) high levels of positive affect, (2) low levels of negative affect, and (3) low levels of perceived stress at t_n–1_.

Third, the effect of initiating an EMI component at t_n–1_ on (1) positive affect, (2) negative affect, and (3) stress at t_n_ was hypothesized to be mediated by an increase in momentary resilience from t_n–1_ to t_n_, while controlling for (1) positive affect, (2) negative affect, and (3) stress at t_n–1_.

## Methods

### Participants and Public Involvement

A convenience sample of participants with a target size of 180 was recruited from the general population and educational counseling services in Germany through a combination of methods, including a call for participation posted on this project’s website and information events held in community settings, such as youth clubs. In addition, after receiving an information workshop on the AI4U training, educational counselors were encouraged to suggest participating in this study to their clients and to integrate their clients’ experiences with the AI4U training in their counseling sessions. With the living lab AI4U, we aimed to incorporate the views of young people through the entire research process and therefore used a peer researcher and four coresearchers from the target population. They actively participated in the design, implementation, and dissemination of this study and especially contributed to the qualitative research conducted as part of the AI4U living lab, which is published elsewhere [35].

### Study Design

The living lab AI4U carried out four transdisciplinary projects involving direct participation of relevant stakeholders, users from the target population, and an interdisciplinary research group. The current study draws on data collected using EMA during three within-subject microrandomized trials (MRT) [36,37] conducted consecutively as part of the second transdisciplinary AI4U project to investigate the effects of applying machine learning (ML) algorithms to deliver EMI components [27]. Findings on the primary outcome of these MRTs and other transdisciplinary projects of the living lab AI4U are published elsewhere [27,35,38,39]. These secondary analyses were preregistered on October 25, 2024, at the Open Science Framework [40] after data collection was completed (June 2024) but before accessing the data (November 2024). Deviations from the preregistration can be found in Multimedia Appendix 2. For reporting this secondary analysis of an MRT, we used the CONSORT (Consolidated Standards of Reporting Trials) reporting checklist [41] and the CONSORT-EHEALTH (Consolidated Standards of Reporting Trials of Electronic and Mobile Health Applications and Online Telehealth) checklist [42], with the latter being included as Checklist 1.

Each participant took part in a 90-minute training session with a coach, that is, a trained study team member who provided guidance on the content and structure of the training before starting the 40-day AI4U training, consisting of a 10-day introductory phase and a 30-day training phase, on a study smartphone. The AI4U training consisted of an EMI administered using a smartphone-based app (movisensXS app, version 1.6.2-beta.4, movisens GmbH) for adaptive real-time and real-world transfer of mental health promotion principles into daily life. During the AI4U training, participants had continuous access to the web-based AI4U dashboard, which displayed summary statistics of the collected EMA data in various charts and tables, and allowed them to self-monitor assessed EMA variables in real time. Members of this study’s team contacted each participant up to two times via phone during the training to address questions and encourage consistent app use. After the AI4U training, participants were invited to a final review session. Data on distal outcomes were collected at baseline and at post intervention. Full details on the procedure and on distal mental health outcomes can be found elsewhere [27].

### Study Setting

The data collection took place at the Central Institute of Mental Health in Mannheim, Germany. Participants were recruited from the general population across Germany and from 5 different public educational counseling services in Baden-Württemberg (Southern Germany).

### Eligibility Criteria

The inclusion criteria were being aged between 14 and 25 years, willingness to participate in the EMI, and ability to give written informed consent independently or through a legal guardian. The exclusion criteria were insufficient command of German, self-reported current diagnosis of any mental health condition, self-reported current psychiatric or psychotherapeutic treatment, and acute suicidality.

All study team members were required to hold at least a bachelor of science in psychology or a related field.

### Ethical Considerations

This study was approved by the Medical Ethics Review Committee II at Heidelberg University (Medical Faculty Mannheim; Ref. No. 2022‐550). Before participation, participants or, for underage participants, their legal guardians gave written informed consent to using the collected data in pseudonymized form for research purposes (see Multimedia Appendix 3). Participation in this study was voluntary, and consent could be withdrawn at any time. Collected data were treated confidentially and were analyzed in a pseudonymized form. Pseudonymized data were stored and processed in accordance with the General Data Protection Regulation. Participants were compensated with €70 (US $81.52) to €120 (US $139.76) for their time, depending on how many times they interacted with the AI4U training. No identification of individual participants in any part of the manuscript or supplementary material is possible.

### Intervention

The AI4U training consisted of a 10-day introductory phase, during which the different EMI components were successively introduced, and a subsequent 30-day training phase. Proximal outcomes were assessed using EMA. This study focuses on the proximal effects of EMI component initiation vs noninitiation on momentary indicators of well-being during the training phase. The main findings of the primary analysis are described elsewhere [27].

The EMI components based on positive refocusing, positive imagery, mindfulness, and compassion-focused techniques consist of four types of exercises: breathing exercises (“counting your breath” and “breathing with breaks”), compass of emotions, positive imagery techniques (“my calm and safe place,” “my compassionate companion,” and “emotion as a wave”), and positive refocusing (“journal of joyful moments” and “positive data log”). A detailed description of the EMI components can be found in Multimedia Appendix 1.

Three types of delivery schemes were used for the delivery of EMI components: enhancing, consolidating, and interactive/adaptive delivery of EMI components, as previously used in other EMIs [24-26,43]. The enhanced delivery of EMI components introduces participants to new EMI components during the introductory phase. The consolidating delivery allows participants to practice previously learned components at user-defined times, both during the introductory phase and the training phase. In addition, during the training phase, the interactive/adaptive delivery scheme provided EMI components after an EMA was completed at a random time. The interactive/adaptive EMI components and the consolidating EMI components during the training phase were provided using repeated randomization with equal probability to either the experimental condition, where one of the seven EMI components was assigned using an ML-algorithm based on EMA ratings of affect and behavior, or the active control condition, where the EMI component was randomly assigned [27,39]. Participants were repeatedly randomized to one of these two conditions up to seven times daily (once for consolidating delivery and six times for interactive/adaptive delivery), resulting in a maximum of 210 decision points. The type of assignment was recorded (ie, random, ML-based, or erroneous), with erroneous occurring in cases where, for example, a technical issue prevented the mobile connection to the server, making an ML-based assignment impossible. While the effect of random vs ML-based assignment was analyzed in the primary analyses, this study only focused on the effect of initiation of EMI components independently of the type of assignment of EMI components. Hence, we controlled for randomization to ML-based assignment of EMI components or random assignment of EMI components in all analyses. As in line with the Multiphase Optimization Strategy [44], the EMI and ML algorithm were optimized after each of the three MRTs; we controlled for the MRT that the participant was assigned to. The optimizations made to the EMI between the MRTs mainly related to the timing of EMA and EMI prompts: there were at least 45 minutes (in MRT 1) or 90 minutes (in MRT 2 and MRT 3) between two EMA prompts and, thus, the subsequent assignment of interactive/adaptive EMI components. These components could be postponed by the participants by up to 20 minutes (in MRT 1) or up to 300 minutes (in MRT 2 and MRT 3). Participants were able to start the EMI component within 50 seconds after it was suggested. When participants did not start the suggested EMI component, they were reminded to do so by an alarm up to 5 times (in MRT 1) or 3 times (in MRT 2 and MRT 3). The consolidating delivery of EMI components occurred twice per day during the introductory phase and once per day during the training phase at a user-defined time and could be postponed by up to 65 minutes (in MRT 1) or 600 minutes (in MRT 2 and MRT 3). In MRT 2 and MRT 3, participants were able to request ML-based assignment of consolidating delivery of EMI components at any time during the day by button press.

### Measures

Sociodemographic data and baseline characteristics were collected with online questionnaires. They included age (in years), gender (male, female, and diverse or not specified), migration history, and psychological distress at baseline, measured by calculating the sum score of the Kessler Psychological Distress Scale (K10) [45].

### Outcomes

During the training phase, EMA included six assessments per day, scheduled at random within set blocks of time at user-defined time periods. Participants received a signal (sound and/or vibration) to complete the EMA. For this analysis, an EMA was considered completed when participants completed the items measuring positive affect, negative affect, stress, and resilience. Momentary positive affect was measured with the following three items: “I feel good,” “I feel relaxed,” or “I feel satisfied.” For momentary negative affect, the three items “I feel scared,” “I feel down,” and “I feel sad” were rated. The mean of the respective items was calculated for positive affect and negative affect, respectively. If any items were missing, the mean was considered missing. Momentary stress was measured using the single item “I feel stressed,” and momentary resilience was measured with the single item “I can handle all the difficulties that I may encounter.” All items were rated on a 7-point Likert scale ranging from not at all (rating of 1) to very much (rating of 7). The primary outcome of this secondary analysis was the level of momentary positive affect, negative affect, and stress at a time point t_n_ in relation to a previous time point t_n–1_, measured using EMA, depending on whether an EMI component was initiated or not initiated by the participant at t_n–1_.

### Harms

Serious adverse events were nonsystematically assessed in the final review session after the AI4U training by asking the participants to describe their experience with the training.

### Sample Size

The required sample size for this secondary analysis was determined based on a study which showed that, to detect a mediation effect with a small effect size (ie, an indirect effect explains at least 4% of the total variance in the outcome variable), the required number of clusters is at least 100 with cluster sizes ranging from 80 to 160 (with power at 80% and *P*<.05) [46]. To detect a medium effect (ie, Cohen *d*=0.5), only 50 clusters would be required (with power at 80% and *P*<.05). In this study, the target size was 180 clusters (ie, participants) with cluster sizes up to 210.

### Randomization and Blinding

Each participant received the same EMI and could decide to initiate or not to initiate an EMI component every time it was suggested automatically by the app after completing an EMA, creating a quasi-experimental “within-subject control condition” (ie, occasions where an EMI component was suggested vs occasions when no EMI component was suggested). Therefore, no randomization of participants to initiation vs noninitiation of an EMI component was performed. This study’s personnel were not blinded.

### Statistical Methods

For the analysis, R (version 4.5.1; R Foundation) was used. To fit linear mixed-effects models, the packages *lme4* (version 1.1.37) [47] and *nlme* (version 3.1.168) [48] were used. For all analyses, the available data were included, and no imputation methods were applied to account for missing data. Little missing completely at random test using the package *naniar* (version 1.1.0) [49] indicated that data were not missing completely at random (*χ*^2^_65_=4729, *P*<.001). We assumed that data were missing at random and therefore used restricted maximum likelihood to estimate our models. Generalized logistic mixed models were fitted to identify covariates associated with missingness, which were included in the models.

All level-1 predictor variables (ie, positive affect, negative affect, stress, and resilience) were centered using person-mean centering and z-standardized. Lagged variables for positive affect, negative affect, and stress were calculated for each time point an EMA prompt was completed, using data from the most recently completed EMA prompt before that time point within the same day. To account for the temporal dependence of the data, within-person residual autocorrelation was modeled using an AR(1) structure for the first and second hypotheses.

To test the first hypothesis, linear mixed-effects models were used to account for the nested structure of EMA data (ie, time points nested within participants). Each model was controlled for age, gender, type of assignment of EMI components, psychological distress at baseline, and assigned MRT. To facilitate the interpretation of results, Cohen *d*-type effect sizes were calculated by dividing the beta coefficient by the square root of the total variance of the model. For the first hypothesis, three separate models were fitted, with the outcome variable well-being at t_n_ being operationalized as (1) positive affect, (2) negative affect, and (3) stress, respectively. The independent variable was the dichotomous variable of whether an EMI component was initiated or not at t_n–1_. It was controlled for (1) positive affect at t_n–1_, (2) negative affect at t_n–1_, or (3) stress at t_n–1_. Random intercepts for individuals were assumed for (1) positive affect at t_n–1_, (2) negative affect at t_n–1_, and (3) stress at t_n–1_ to allow for interindividual differences in the level of well-being. Random slopes for the effects of well-being at t_n–1_ and initiation of an EMI component at t_n–1_ were initially assumed, but the models were revised to exclude the random slope for well-being at t_n–1_ due to convergence issues.

For the second hypothesis, a moderator variable was added to the model. The moderator was the lagged well-being variable (1) positive affect at t_n–1_, (2) negative affect at t_n–1_, or (3) stress at t_n–1_. It was included in the model as a two-way interaction term with the dichotomous variable indicating whether an EMI component was initiated at t_n–1_. Random intercepts for the individuals were assumed for (1) positive affect at t_n–1_, (2) negative affect at t_n–1_, and (3) stress at t_n–1_ to allow for interindividual differences in the level of well-being. In addition, random slopes for the effects of well-being at t_n–1_ and initiation of an EMI component at t_n–1_, and the interaction of well-being at t_n–1_ and initiation of an EMI component at t_n–1_ were initially assumed, but due to convergence issues, the models were revised, including only random slopes for initiation of an EMI component at t_n–1_. To investigate whether there were differences in the magnitude of the effect of EMI initiation at t_n–1_ on well-being at t_n_ within occasions with high (mean+1 SD) vs low (mean −1 SD) levels of well-being at t_n–1_, linear combinations of coefficients were calculated using the R package *multcomp* [50], with well-being indicators entered as continuous variables in the model.

For the third hypothesis, three mediation models were fitted with difference scores (t_n_ – t_n–1_) for the well-being variables (1) positive affect, (2) negative affect, and (3) stress as the outcome variables and the difference score (t_n_ – t_n–1_) for resilience as the mediator. To examine the mediation effects, a two-step multilevel modeling approach was used: first, a linear mixed-effects model was fitted to predict the mediator (ie, the change in resilience) depending on whether an EMI component was initiated at t_n–1_. Second, a linear mixed-effects model was fitted to predict the outcome (ie, the change in well-being) depending on the initiation of an EMI component at t_n–1_ and the difference score for resilience. The initial model specification included random slopes for both the mediator and the outcome model. However, the models did not converge properly, and parameter estimates suggested that the random slopes did not explain meaningful variability. To ensure model stability and interpretability, the model was specified, including only random intercepts for the individuals. To test the mediation effect of the change in resilience on the relationship between initiation of an EMI component and the change in well-being, the function “mediate” from the R package *mediation* (version 4.5.1) [51] was used. The mediation effect was evaluated using 1000 bootstrap simulations to estimate indirect, direct, and total effects, along with their statistical significance.

Two sensitivity analyses will be conducted for all analyses: first, we will investigate whether the results remain the same when considering the specific type of EMI component (vs not initiating any EMI component). The type of EMI component (including no EMI component) will be operationalized as a categorical predictor in the models. Second, the reason why an EMI component was not initiated (ie, because none was suggested by the app vs because the participant did not initiate the suggested component) will be considered. The reason for noninitiation will be included as a categorical predictor.

## Results

### Participant Flow

**Figure 1.**
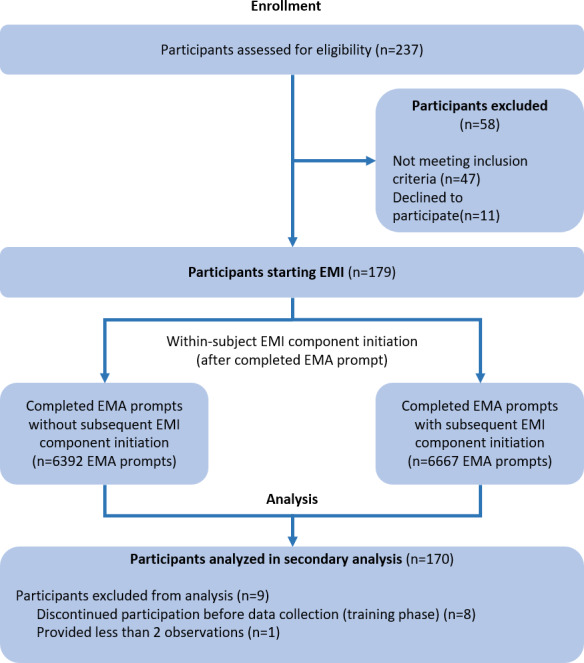
Flow diagram of participants according to CONSORT guidelines [41]. The flowchart outlines participant inclusion in (a) the AI4U study and (b) the EMI, after which each participant could repeatedly initiate or not initiate a suggested EMI component after the completion of an EMA prompt. AI4U: artificial intelligence for personalized digital mental health promotion in youth; CONSORT: Consolidated Standards of Reporting Trials; EMA: ecological momentary assessment; EMI: ecological momentary intervention.

Of the 408 individuals identified for potential participation, 237 could be screened for eligibility. Of those, 190 individuals met the inclusion criteria and provided written informed consent, but 11 participants discontinued their study participation before starting the AI4U training. The full sample of participants that started the AI4U training, therefore, consisted of 179 participants. Of those, 8 participants did not continue with the training phase after the 10-day introductory phase, and 1 participant completed less than two EMA prompts within any day, and their data thus were not included in the analyses. Therefore, the analytic sample for this analysis comprised 170 individuals (ie, 56 in MRT 1, 56 in MRT 2, and 58 in MRT 3) recruited from the general population (n=156) and educational counseling services (n=14). The participant flow is depicted in Figure 1 according to the CONSORT guidelines [41]. The recruitment and the subsequent data collection took place between July 2022 and June 2024.

### EMI Delivery

During the 30-day training phase of the EMI, both EMA prompts and EMI components were presented to the participants. In total, participants completed 13,059 EMA prompts and missed or did not complete 16,802 EMA prompts, which leads to a completion rate of 43.7% (13,059/29,861). The time of day did not have a significant effect on the completion of an EMA prompt (vs noncompletion); however, the day of training since starting the training phase did have a small effect on the completion of an EMA prompt (Multimedia Appendix 4). Within the same day, the mean time interval between two completed EMA prompts was 178.8 (SD 135.4) minutes, and the mean time interval between an initiated EMI and the next consecutive completed EMA prompt was 167.0 (SD 115.4) minutes. In response to 51.1% (6667/13,059) of the completed EMA prompts, interactive/adaptive EMI components were initiated within 45 minutes after the EMA was completed. The frequency distribution for initiating EMI components was broadly similar across the eight different EMI components (see Multimedia Appendix 5).

### Baseline Data and Sample Characteristics

The analytic sample was mostly female (125/170, 73.5%), with a mean age of 20.41 (SD 3.40) years. Characteristics of the full sample and the analytic sample, as well as aggregated means, skewness, and kurtosis of the proximal outcome variables, can be found in Table 1. The Spearman’s rank correlations of the person means of the proximal outcome variables with psychological distress and resilience measured at baseline are reported in Multimedia Appendix 6. No serious adverse events, privacy breaches, or unintended effects were detected during this study.

**Table 1.** Sample characteristics (gender, age, migration history, psychological distress, etc) of the full sample and the analytic sample, and descriptive statistics of proximal outcomes assessed by EMA (positive affect, negative affect, stress, and resilience) and time intervals between EMA and EMI of the analytic sample.

Sample characteristics		Full sample (n=179)	Analytic sample (n=170)
Gender, n (%)^a^			
Female		128 (71.5)	125 (73.5)
Male		44 (24.6)	39 (22.9)
Diverse or not specified		3 (1.7)	3 (1.8)
Age (years), mean (SD)		20.36 (3.39)	20.41 (3.40)
Migration history, n (%)^a^			
No migration history		83 (46.4)	79 (46.5)
Foreign born		10 (5.6)	9 (5.3)
First-generation migrant		48 (26.8)	46 (27.1)
Second-generation migrant		25 (14)	24 (14.1)
Psychological distress at baseline, n (%)^a^^,^^b^			
None		50 (27.9)	49 (28.8)
Mild		60 (33.5)	58 (34.1)
Moderate		37 (20.7)	34 (20)
Severe		25 (14)	23 (13.5)
Descriptive statistics of proximal outcomes assessed by EMA^c^^,^^d^			
Positive affect			
Mean (SD)		—^e^	4.65 (1.17)
Skewness		—	−0.47
Kurtosis		—	0.02
Between-person reliability^f^		—	0.96
Within-person reliability^f^		—	0.78
Negative affect			
Mean (SD)		—	2.15 (1.13)
Skewness		—	1.38
Kurtosis		—	1.88
Between-person reliability^f^		—	0.93
Within-person reliability^f^		—	0.70
Stress			
Mean (SD)		—	3.03 (1.66)
Skewness		—	0.82
Kurtosis		—	−0.13
Resilience			
Mean (SD)		—	4.73 (1.35)
Skewness		—	–0.52
Kurtosis		—	0.03
Descriptive statistics on the time interval between completed EMA and initiated EMI			
Time in minutes between two consecutive completed EMAs within the same day			
Mean (SD)		—	178.8 (135.4)
Median (IQR)		—	140.1 (91.7-218.7)
Time in minutes between an initiated EMI and the consecutive completed EMA within the same day			
Mean (SD)		—	167.0 (115.4)
Median (IQR)		—	134.6 (91.9-202.9)

a Number of participants with missing data, n (%), full sample/analytic sample: gender 4 (4/179, 2.2%)/3 (3/170, 1.8%), age 4 (4/179, 2.2%)/3 (3/170, 1.8%), migration history 13 (13/179, 7.3%)/12 (12/170, 7.1%), and psychological distress 7 (7/179, 3.9%)/6 (6/170, 3.5%).

b Cutoffs to categorize psychological distress measured with K10: ”none” (range score: 10 to 19); “mild” (range score: 20-24); “moderate” (range score: 25-29); “severe” (range score: 30-50).

c EMA: ecological momentary assessment.

d All EMA items have been completed on a 7-point scale (1=not at all, 7=very much). All values (except reliability) represent aggregated values over the 30-day training phase.

e Not available.

f Reliabilty was calculated by applying the function “compRelSem” of the R package *semTools* (version 0.5-7) [52] after estimating a confirmatory factor analysis of the 3-item scales with participants as clusters using *lavaan *(version 0.6.20) [53,54].

### Initiation of an EMI Component at t_n–1_ and Well-Being at t_n_

To examine whether well-being differed at t_n_ depending on whether or not an EMI component was initiated after the previous EMA prompt, linear mixed-effects models were specified for each of the three proximal outcome variables (ie, positive affect, negative affect, and stress). All outcomes at t_n–1_ were associated with the respective outcome at t_n_ (Table 2). Findings did not signal a difference in positive affect at t_n_ (*b*=0.00, 95% CI −0.04 to 0.04, *d*=0.00), negative affect at t_n_ (*b*=−0.03, 95% CI −0.06 to 0.01, *d*=−0.03), or stress at t_n_ (*b*=0.01, 95% CI −0.03 to 0.05, *d*=0.01) for initiating an EMI component at t_n–1_ vs not initiating an EMI component at t_n–1_, as zero was included in the 95% CI. None of the potential confounding variables was associated to a relevant extent with the proximal outcomes. Sensitivity analyses showed that the results remained largely unchanged when analyzing the effect of initiating a specific type of EMI component vs not initiating any EMI component (Multimedia Appendix 7). The reason why an EMI component was not initiated also did not alter the results (Multimedia Appendix 8).

**Table 2.** Regression coefficients, 95% CIs, *P* values, and effect sizes of the linear mixed model for the effect of initiating an EMI^a^ component at the previous time point, t_n–1_, on well-being at the later time point t_n_^b^.

	*b* value	95% CI	*P* value	Cohen *d*
Outcome: positive affect at t_n_				
Initiation of EMI component at t_n–1_	0.00	−0.04 to 0.04	.96	0.00
Positive affect at t_n–1_	0.44	0.42 to 0.46	<.001	0.50
Outcome: negative affect at t_n_				
Initiation of EMI component at t_n–1_	−0.03	−0.06 to 0.01	.17	−0.03
Negative affect at t_n–1_	0.41	0.39 to 0.43	<.001	0.46
Outcome: stress at t_n_				
Initiation of EMI component t_n–1_	0.01	−0.03 to 0.05	.60	0.01
Stress at t_n–1_	0.39	0.37 to 0.41	<.001	0.42

a EMI: ecological momentary intervention.

b Adjusted for potential confounding by age, gender, assignment of ecological momentary intervention component, microrandomized trial, and psychological distress at baseline.

### Effects of Initiating an EMI Component at t_n–1_ on Well-Being at t_n_ by Well-Being at t_n–1_

Linear mixed-effects models were used to examine whether the magnitude of the effect of initiating an EMI component at t_n–1_ vs not initiating an EMI component at t_n–1_ on well-being at t_n_ differed depending on the level of well-being at t_n–1_ (Table 3). When taking multiple testing into account, there was no strong difference in the magnitude of the effect of initiating an EMI component vs not initiating an EMI component at t_n–1_ on (1) positive affect at t_n_ when positive affect at t_n–1_ was high (mean +1 SD) vs low (mean −1 SD, *b*=0.08, 95% CI 0.01 to 0.16, *d*=0.09), (2) negative affect at t_n_ when negative affect at t_n–1_ was high vs low (*b*=−0.09, 95% CI −0.17 to −0.01, *d*=−0.10), (3) stress at t_n_ when stress at t_n–1_ was high vs low (*b*=0.09, 95% CI 0.01 to 0.17, *d*=0.10 (with the effect sizes being small or falling just short of being small. Also, findings signaled trivial effect sizes for (1) the difference in positive affect at t_n_ for initiating vs not initiating an EMI component at t_n–1_ when positive affect at t_n–1_ was high (*d*=0.05) and low (*d*=−0.04), (2) the difference in negative affect at t_n_ for initiating vs not initiating an EMI component at t_n–1_ when negative affect at t_n–1_ was high (*d*=−0.08) and low (*d*=0.02), and (3) the difference in stress at t_n_ for initiating vs not initiating an EMI component at t_n–1_ when stress at t_n–1_ was high (*d*=0.06) and low (*d*=−0.04). All outcome variables at t_n–1_ were associated with the respective outcome variables at t_n_ (Table 3). Sensitivity analyses revealed that the results remained broadly similar when considering the type of EMI component (Multimedia Appendix 7) and the reason for noninitiation of an EMI component into account (Multimedia Appendix 8).

**Table 3.** Regression coefficients, 95% CIs, *P* values, and effect sizes of the linear mixed models for the effect of initiating an EMI^a^ component at the previous time point t_n–1_ on well-being at the later time point t_n_ by well-being at t_n–1_^b^, with linear comparisons of coefficients for high (mean +1 SD) vs low (mean −1 SD) levels of well-being at t_n–1_^b^.

	*b* value	95% CI	*P* value	Cohen *d*
Outcome: positive affect at t_n_				
Initiation of EMI component at t_n–1_	0.00	−0.04 to 0.04	.92	0.00
Positive affect at t_n–1_	0.42	0.40 to 0.45	<.001	0.48
Initiation of EMI component at t_n–1_ × positive affect at t_n–1_	0.04	−0.00 to 0.08	.04	0.05
High positive affect	0.04	−0.02 to 0.10	.26	0.05
Low positive affect	−0.04	−0.10 to 0.02	.30	−0.04
High vs low positive affect	0.08	0.01 to 0.16	.04	0.09
Outcome: negative affect at t_n_				
Initiation of EMI component	−0.03	−0.06 to 0.01	.17	−0.03
Negative affect at t_n–1_	0.43	0.41 to 0.46	<.001	0.49
Initiation of EMI component at t_n–1_ × negative affect at t_n–1_	−0.05	−0.08 to -0.01	.02	−0.05
High negative affect	−0.70	−0.14 to -0.01	.02	−0.08
Low negative affect	0.02	−0.04 to 0.08	.75	0.02
High vs low negative affect	−0.09	−0.17 to -0.01	.02	−0.10
Outcome: stress at t_n_				
Initiation of EMI component t_n–1_	0.01	−0.03 to 0.05	.63	0.01
Stress at t_n–1_	0.36	0.34 to 0.40	<.001	0.40
Initiation of EMI component at t_n–1_ × stress at t_n–1_	0.05	−0.01 to 0.09	.02	0.05
High stress	0.06	−0.01 to 0.12	.11	0.06
Low stress	−0.04	−0.10 to 0.03	.39	−0.04
High vs low stress	0.09	0.01 to 0.17	.02	0.10

a EMI: ecological momentary intervention.

b Adjusted for potential confounding by age, gender, assignment of ecological momentary intervention component, microrandomized trial, and psychological distress at baseline.

### Effects of Initiating an EMI Component at t_n–1_ on Changes in Well-Being From t_n–1_ to t_n_ via Changes in Resilience From t_n–1_ to t_n_

Three mediation analyses were conducted to examine the effects of initiation of an EMI component on changes in proximal well-being outcomes from t_n–1_ to t_n_ via changes in momentary resilience from t_n–1_ to t_n_ as a potential mediator while controlling for well-being at t_n–1_. The results of the linear mixed models for the mediator indicated that there was no difference in change in resilience from t_n–1_ to t_n_ for initiating an EMI component at t_n–1_ vs not initiating an EMI component at t_n–1_ (Multimedia Appendix 9). Change in resilience was associated with changes in well-being in all three models (Multimedia Appendix 9): change in resilience predicted change in positive affect (*b*=0.22, 95% CI 0.21 to 0.24, *d*=0.27), indicating that a greater increase in resilience was associated with a greater increase in positive affect. Similarly, change in resilience predicted change in negative affect (*b*=−0.16, 95% CI −0.18 to −0.15, *d*=−0.19), suggesting that a greater increase in resilience was associated with a greater decrease in negative affect. Change in resilience was also a predictor of change in stress (*b*=−0.15, 95% CI −0.17 to −0.13, *d*=−0.14), indicating that an increase in resilience was associated with a decrease in stress. In all three models, the effect of initiating an EMI component on changes in proximal well-being outcomes was not mediated via changes in resilience (Table 4). Findings were broadly similar in sensitivity analyses taking into account the specific type of EMI component (Multimedia Appendix 7) and the reason for not initiating an EMI component (Multimedia Appendix 8).

**Table 4.** Regression coefficients, 95% CIs, and *P* values of the mediation model for the effect of initiating an EMI^a^ component at t_n–1_ on change in well-being from t_n–1_ to t_n_ via change in resilience from t_n–1_ to t_n_^b^.

	*b* value	95% CI	*P* value
Outcome: change in positive affect			
Total effect	0.00	−0.03 to 0.04	.91
Direct effect	−0.00	−0.04 to 0.03	.86
Indirect effect	0.00	−0.01 to 0.02	.36
Proportion mediated	0.10	−4.69 to 4.35	.84
Outcome: change in negative affect			
Total effect	−0.03	−0.06 to 0.01	.16
Direct effect	−0.02	−0.06 to 0.01	.21
Indirect effect	−0.00	−0.01 to 0.01	.51
Proportion mediated	0.09	−0.80 to 1.17	.55
Outcome: change in stress			
Total effect	0.01	−0.02 to 0.05	.50
Direct effect	0.01	−0.02 to 0.05	.50
Indirect effect	0.00	0.00 to 0.00	>.99
Proportion mediated	0.00	0.00 to 0.00	>.99

a EMI: ecological momentary intervention.

b Adjusted for potential confounding by age, gender, assignment of ecological momentary intervention component, microrandomized trial, and psychological distress at baseline.

## Discussion

### Principal Findings

This secondary analysis of data collected in the AI4U project sought to explore proximal effects of initiating EMI components of a compassion-focused smartphone-based digital training for youth on momentary outcomes of well-being (ie, positive affect, negative affect, and stress) assessed at the next time point using EMA. We did not find convincing evidence that well-being at a time point improves compared to the previous time point, depending on whether an EMI component was initiated at the previous time point vs when no EMI component was initiated. There was also no strong indication that this potential effect may be influenced by the level of well-being at the time of EMI initiation. While changes in resilience predicted changes in well-being, the results did not suggest that the effect of initiating an EMI component on mental well-being may be partially mediated by an increase in momentary resilience.

### Interpretation

There is growing evidence on the effectiveness of EMI for various outcomes related to mental health [28-30]. However, many previous studies [20,21,28-30] and also the primary analysis of the current study [27] focused on distal outcomes in promoting mental health and well-being. While evidence on these outcomes is important, they are more limited in how exactly specific EMI components exert their effects over time [31,32]. Our study extended previous research by investigating proximal effects of EMI and thereby addressing the theoretical assumption [19], building on prior evidence [20,21,55], that EMIs have an effect at the momentary level. However, so far, there is limited evidence on proximal effects of EMIs on mental health–related outcomes: in one study, by Bernstein et al [56], an EMI was used to promote CBT skills practice in patients admitted for suicidal ideation or behavior during and after their inpatient stay. It showed that when an EMA measuring negative affect was followed by an EMI to engage in CBT skills practice, negative affect measured directly after the skills practice improved compared to when no EMI was prompted [56]. While the sample was small (n=25) and drawn from a specific clinical population, the results highlight that the immediate effects of an EMI can be detected using EMA [56]. Nevertheless, the authors emphasized that as the current study lacked a control group, it may be possible that the observed effects were not exclusively attributable to the EMI, but rather influenced by the multiple sessions conducted during the inpatient stay, in which participants learned CBT skills [56]. In contrast to this study, in our study, the subsequent EMA measuring positive and negative affect and stress did not differ depending on whether a single EMI component was initiated vs not initiated at the previous time point, and the next EMA did not immediately follow the EMI component. In addition, our sample consisted of young individuals who were not necessarily in contact with mental health services and without a current diagnosis of a mental health condition. The EMI implemented in this study did, accordingly, not serve as a therapeutic intervention but an intervention for mental health promotion and prevention. Hence, our sample is also likely to have experienced a lower intensity or fewer fluctuations of negative affective states [57,58]. This assumption is supported by the distribution of ratings of EMA items (Table 1), which, for all proximal outcome variables, is skewed toward ratings indicating high well-being (ie, low stress, low negative affect, and high positive affect), indicating potential floor and ceiling effects.

In a different study by Vinci et al [32], an EMI for smoking cessation was found to have proximal effects on negative affect at the next timepoint as well as after completion of one week of the training. However, the effect of the EMI could not be observed on other outcome variables (eg, positive affect) [32]. Therefore, while the study used a different intervention and focused on a different population [32], the results align, to some extent, with the results of the current study. In the primary analysis of this study, using data from the first of three MRTs, the authors found that when EMI components were allocated ML-based, levels of momentary resilience at the next time point were, on average, higher compared to when EMI components were allocated at random [27]. In addition, they found evidence for uncontrolled effects of the EMI on distal outcomes, that is, an increase in resilience and the use of adaptive emotion regulation strategies and a decrease in psychological distress [27]. Furthermore, qualitative analyses of the AI4U training indicate that the participants’ subjective mental well-being did improve during the AI4U training, as several participants reported improvements in their emotion regulation and self-reflection on emotions [35].

Echoing previous research [6,7], our results showed that resilience was associated with indicators of well-being. This highlights the role resilience may play for mental well-being [6,7]. Previous research on the relationship between resilience and well-being has primarily relied on cross-sectional assessments [7]. With our study, we expand this evidence by investigating the association between changes in momentary resilience, on the one hand, and changes in momentary affect and stress, on the other. Our results showed that an increase in resilience from one EMA to the next was associated with an increase in positive affect and a decrease in negative affect and stress. This finding does not allow us to draw conclusions on a causal relationship between changes in resilience and changes in well-being: an increase in resilience may lead to an increase in well-being, but also increased well-being may lead to more positive ratings of resilience [59]—or changes in both may be due to another, unobserved variable. The findings do, however, indicate that the widely reported association of resilience and well-being in youths and adolescents [6,7] can also be found at the momentary level. Momentary resilience was, however, not affected by the initiation of an EMI component at the previous time point. One possible explanation for this may be that, for digital interventions to be effective, it is necessary that participants engage with them [60,61].

The high variability in and mean number of days of training in this sample (Table 1) may indicate that, while many participants strongly engaged with the training, some engaged only to a limited extent. The duration of this study allowed the overall number of completed EMA prompts representing the proximal outcome of this study to be high (n=13,059). However, 56.3% (16,802/29,861) of EMA prompts were not completed or missed. While we assume that noncompletion of EMA prompts occurred at random and have verified that it is not associated with the time of day (see Multimedia Appendix 4), it is still possible that the available data may be biased due to the participants only responding to the EMA prompts in certain moments. We observed that the likelihood of not completing an EMA prompt increased in the course of the training (Multimedia Appendix 4), indicating that overall, participants tended to engage less with the EMI as the training progressed. According to qualitative feedback of participants, possible reasons for low engagement could be feeling uncomfortable using the AI4U training in public or experiencing the repeated prompts as distracting [35]. As low compliance may reduce exposure to EMI components over time [60,61], it may attenuate its potential impact: Without sufficient interaction with the EMI, the mechanisms leading to an increase in resilience and well-being may not be active to a detectable degree. Thus, the lack of proximal effects of EMI initiation on the next EMA may not necessarily indicate that there is no proximal effect of the EMI, but may rather be due to low adherence to the EMI. Thus, identifying factors associated with low adherence should be the focus of further research [32].

Another explanation for our findings may be that the completion of EMA prompts may have, per se, an effect on well-being and, hence, reflected an active “within-subject control condition” noninferior to proximal effects of EMI components. Prior research has shown that smartphone-based self-monitoring of emotions can have a positive effect on mental health even without the implementation of an EMI [62,63]. The potential mechanism behind this effect may be that emotional self-awareness may increase as a result of repeated self-monitoring [62], which, in turn, may positively impact mental health [62]. It is tempting to speculate whether the EMA delivered as part of the AI4U training may have had an effect on well-being that obscured the effect of the EMI. However, the design of this study does not allow for disentangling this potential masking effect, which highlights the importance of future methodological work on how EMI operates and exerts their beneficial effects.

### Limitations

When interpreting the reported results, it is important to consider that all analyses were performed as secondary analyses and that the original study [27] was neither designed nor powered to test the hypotheses of this paper. Sample size considerations for these secondary analyses were based on a previous simulation study [46], which indicates that the available sample size may be sufficient for these secondary analyses. However, it is possible that the sample size was still not large enough to detect effects of EMI component initiation, taking into account that each of the three MRTs was primarily powered to detect one proximal effect of ML-based EMI delivery and not for additional tests performed as part of current exploratory analyses [27]. In addition, the following five methodological aspects need to be considered.

First, to explore proximal effects of EMI components in the current analysis, completed EMA prompts before (t_n–1_) and after (t_n_) EMI components that were initiated were considered. The time interval between an EMI and the next completed EMA prompt was not fixed and depended on the compliance of participants. Thus, it may be the case that participants missed or ignored EMA prompts after initiation of the EMI. The subsequent EMA prompt was therefore not necessarily in a close temporal relationship with the preceding EMI component. In the time interval between an EMI component and the following EMA, proximal effects may have attenuated and/or external influences may have occurred with a potentially larger effect on affect and stress than the EMI component. While the current approach allowed for some approximation to proximal effects of EMI components, a change in affect or stress from t_n–1_ to t_n_ does not depict the actual proximal effect of EMI components, as in some cases, several hours could have passed between initiating the EMI component and completing the next EMA prompt. Thus, future studies on proximal effects of EMI components should implement an EMA prompt directly after each EMI component (ie, within minutes).

Second, due to the nature of EMA and EMI, participants completed both the assessments and the interventions in their daily life, that is, without supervision [15,18]. Therefore, it remains unknown whether the EMI component that was initiated was indeed completed in accordance with the instructions or participants were distracted by, or occupied with other tasks [64]. Qualitative data shows that several participants found it challenging to complete EMA and EMI when not at home, but, for example, in public, and that participants were sometimes not conscientious [35]. Likewise, participants were provided with the option to perform EMI exercises that they found helpful on their own (as part of consolidating delivery of EMI components), that is, when no EMI component was suggested by the app. It is also possible that participants performed exercises they learned during the AI4U training independently, as several participants reported that they integrated the breathing intervention into their everyday lives even beyond the duration of the study [35]. This may have masked potential effects of the EMI component and thus biased our findings. Asking participants, for example, as part of the EMA for collecting proximal outcome data or at the end of the EMI training phase, to which degree they complied with the training instructions or implemented EMI exercises into their everyday lives independent of being prompted, may provide more insight into EMI adherence, engagement, and fidelity [65].

Third, this analysis focused on the effects of EMI components that were initiated after EMA completion upon suggestion (ie, interactive/adaptive EMI), which allowed for approximating a quasi-experimental “within-subject control condition” [26] (ie, occasions where an EMI component was suggested vs occasions when no EMI component was suggested). However, the sampling scheme was more complex, including consolidating delivery of EMI components on demand and at prescheduled times based on self-set reminders [27]. Especially, self-initiated EMI components may be very likely to be completed according to instructions, with high fidelity, when deemed helpful by participants. Therefore, especially self-initiated EMI components may have a more marked effect on mental well-being. Future investigations should therefore consider all initiated EMI components in investigating proximal EMI effects, including self-initiated EMI components.

Fourth, the EMI components differed in their content, and not all EMI components were designed and expected to generate immediate effects. For example, the “compass of emotions” component served predominantly psychoeducational purposes that may be unlikely to have a proximal effect. When looking at each of the types of EMI components separately, sensitivity analysis did not detect consistent effects of the type of EMI that was initiated. This contrasts with the findings of other authors [34], who found initial signals that the effect of completing a compassion-focused EMI component (vs not completing an EMI component) was more pronounced when the type of EMI component was either a “breathing exercise” or “soothing imagery” (comparable to “positive imagery” in this study). It may therefore be valuable to further investigate the effects of different EMI types.

Fifth, to keep the burden as low as possible for participants [64], while still ensuring that all relevant aspects were assessed, proximal outcomes were assessed with only one to three items. However, this may result in only a limited aspect of the broader constructs of affect, stress, and resilience being captured. Especially resilience, which is assessed in this study using only a single item, is thought to be actually a very complex and dynamic process comprising various resilience factors [66,67]. The item used in this study, “I can handle all the difficulties that I may encounter,” was intended to capture the capacity to withstand or recover quickly from difficulties, as a core aspect of resilience [66], but it neglects other important aspects such as perceived social support and sense of coherence [68,69]. A more comprehensive EMA measure of resilience may facilitate the detection of changes in these specific aspects, but it needs to be carefully considered in the light of the additional burden on the participants.

### Conclusions

This secondary analysis did not support the hypothesis that momentary mental well-being (ie, positive affect, negative affect, or stress) at a time point differed depending on whether or not an EMI component was initiated at the previous time point during a 30-day EMI training for mental health promotion in youths. To our knowledge, this is the first study to investigate proximal effects of components of a compassion-focused EMI for mental health promotion on momentary positive affect, negative affect, and stress in a nonclinical sample of youths. This study thereby expands existing research on the effects of EMI by investigating proximal effects of EMI components on momentary outcomes of mental well-being using EMA and by presenting the innovative approach to investigate potential mechanisms of how EMI leads to long-term improvements of distal outcomes. This can serve as a basis for future research on momentary effects of EMI components, which should consider several aspects relevant for disentangling beneficial proximal and distal effects of EMIs, as reported by prior research [27], such as the implementation of more comprehensive instruments to assess proximal outcomes in closer temporal proximity to the previous EMI component and the importance of compliance with the EMI. Building on and expanding the approach and results presented in this secondary analysis can allow us to further extend our knowledge on proximal effects of EMI and to identify relevant factors for EMI to be effective in supporting mental well-being. Understanding more fully the proximal effects of EMI and how they unfold to translate into previously reported beneficial effects on distal outcomes has the potential to support the development of scalable digital interventions and thereby improve the public’s mental health and well-being.

## Supplementary material

10.2196/85552 Multimedia Appendix 1 Description of the ecological momentary intervention components.

10.2196/85552 Multimedia Appendix 2 Deviations from the preregistration.

10.2196/85552 Multimedia Appendix 3 Informed consent form for adult participants.

10.2196/85552 Multimedia Appendix 4 Effect of time of day and day of training on noncompletion vs completion of an ecological momentary assessment prompt.

10.2196/85552 Multimedia Appendix 5 Characteristics of the 30-day training phase.

10.2196/85552 Multimedia Appendix 6 Spearman rank correlations of variables measured using ecological momentary assessment with psychological distress and resilience at baseline.

10.2196/85552 Multimedia Appendix 7 Sensitivity analyses for type of ecological momentary intervention component.

10.2196/85552 Multimedia Appendix 8 Sensitivity analysis for reason of ecological momentary intervention component noninitiation.

10.2196/85552 Multimedia Appendix 9 Mediator and outcome model for hypothesis 3.

10.2196/85552 Checklist 1 CONSORT-EHEALTH checklist.

## Abbreviations

AI4U: artificial intelligence for personalized digital mental health promotion in youth
CBT: cognitive behavioral therapy
CONSORT: Consolidated Standards of Reporting Trials
CONSORT-EHEALTH: Consolidated Standards of Reporting Trials of Electronic and Mobile Health Applications and Online Telehealth
EMA: ecological momentary assessment
EMI: ecological momentary intervention
K10: Kessler Psychological Distress Scale
mHealth: mobile health
ML: machine learning
MRT: microrandomized trial
RCT: randomized controlled trial
