# Supplementary material for: Promoting Psychological Resilience and Well-Being in Youth With a Smartphone-Based Ecological Momentary mHealth Intervention: Secondary Analysis of a Microrandomized Trial
Source: J Med Internet Res. 2026 Jun 18;28:e85552. doi: 10.2196/85552 (PMC13280375; doi:10.2196/85552)
Supplement: Multimedia Appendix 1 [file jmir-v28-e85552-s001.docx]

1. Compass of emotions

The compass of emotion has several emotional dimensions such as danger, calmness, safety, goals and needs. Individuals are encouraged to rate the intensity of their emotions and investigate potential triggers.

1. Counting your breath

Individuals are asked to count to 5 between inhaling and exhaling.

1. My calm and safe place

Individuals are guided to imagine a peaceful place and to explore this place in their mind.

1. Breathing with breaks

Individuals are asked to focus on the transition from inhaling to exhaling.

1. My compassionate companion

Individuals are encouraged to image an unpleasant situation and to imagine how a compassionate companion that knows and understands them well would react and support them.

1. Emotion as a wave

Emotions are compared to waves in the sea that come and go. Individuals are encouraged to recall a past unpleasant situation and „surf“ the waves of emotions instead of being overwhelmed by them.

1. Journal of joyful moments

Individuals are asked to note small joyful moments that they experience in daily life

1. Positive data log

Individuals are encouraged to note down successes that they experienced in their everyday life.
