# Supplementary material for: Promoting Psychological Resilience and Well-Being in Youth With a Smartphone-Based Ecological Momentary mHealth Intervention: Secondary Analysis of a Microrandomized Trial
Source: J Med Internet Res. 2026 Jun 18;28:e85552. doi: 10.2196/85552 (PMC13280375; doi:10.2196/85552)
Supplement: Multimedia Appendix 2 [file jmir-v28-e85552-s002.docx]

Wierzba E, Schick A, Rauschenberg C, Fechtelpeter J, Hiller S, Götzl C, Durstewitz D, Krumm S, Koppe G, Reininghaus U. Promoting psychological resilience and well-being in youth with a smartphone-based ecological momentary mHealth intervention. Open Science Framework - Preregistration. 2024; Available from: https://osf.io/738u5/?view_only=1745d36e147d497e919d5f9ed8755dc1.

- Psychological distress at baseline was added as an additional covariate in all models, as we found upon suggestion of a reviewer that psychological distress at baseline predicted a lower completion rate.
- Sensitivity analyses were conducted for a) type of EMI component and b) for reason of EMI component non-initiation as new evidence of a similar study [1] suggested that these factors may influence the results.
- Sensitivity analyses were conducted for baseline psychological distress as an additional covariate upon reviewer’s request.
- Instead of performing exploratory sensitivity analyses for age, gender, assignment of EMI component and assignment of MRT, those variables were included in the models as control variables. This was done so that potential effects of the control variables could be regarded in all analyses.
- Cohen’s d-type effect sizes were calculated in addition to 95% confidence intervals of the regression coefficients and *P*-values to improve the interpretability of the results.
- In addition to person-mean centering, we used z-standardization for the EMA measures, which was necessary to interpret the effects of the interaction models.

# References

1. Gugel J, Paetzold I, Rauschenberg C, Schick A, Hirjak D, Boehnke JR, et al. Zooming in: Proximal effects of a transdiagnostic, ecological momentary intervention for enhancing resilience in help-seeking young people (EMIcompass). JMIR Preprints. Preprint posted online May 08, 2025. doi: 10.2196/preprints.77150.
