# Supplementary figures and images for: Promoting Psychological Resilience and Well-Being in Youth With a Smartphone-Based Ecological Momentary mHealth Intervention: Secondary Analysis of a Microrandomized Trial
Source: J Med Internet Res. 2026 Jun 18;28:e85552. doi: 10.2196/85552 (PMC13280375; doi:10.2196/85552)

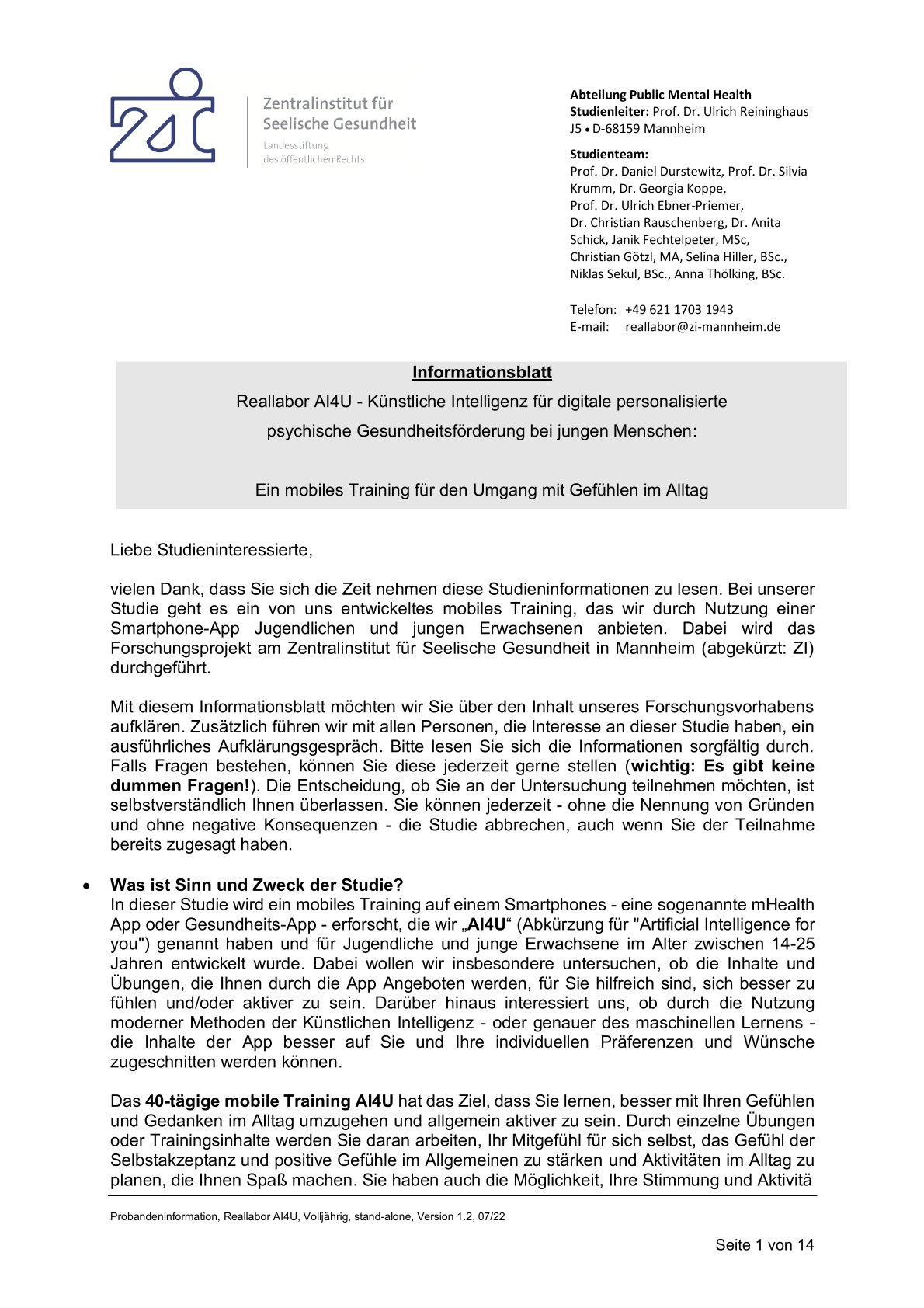


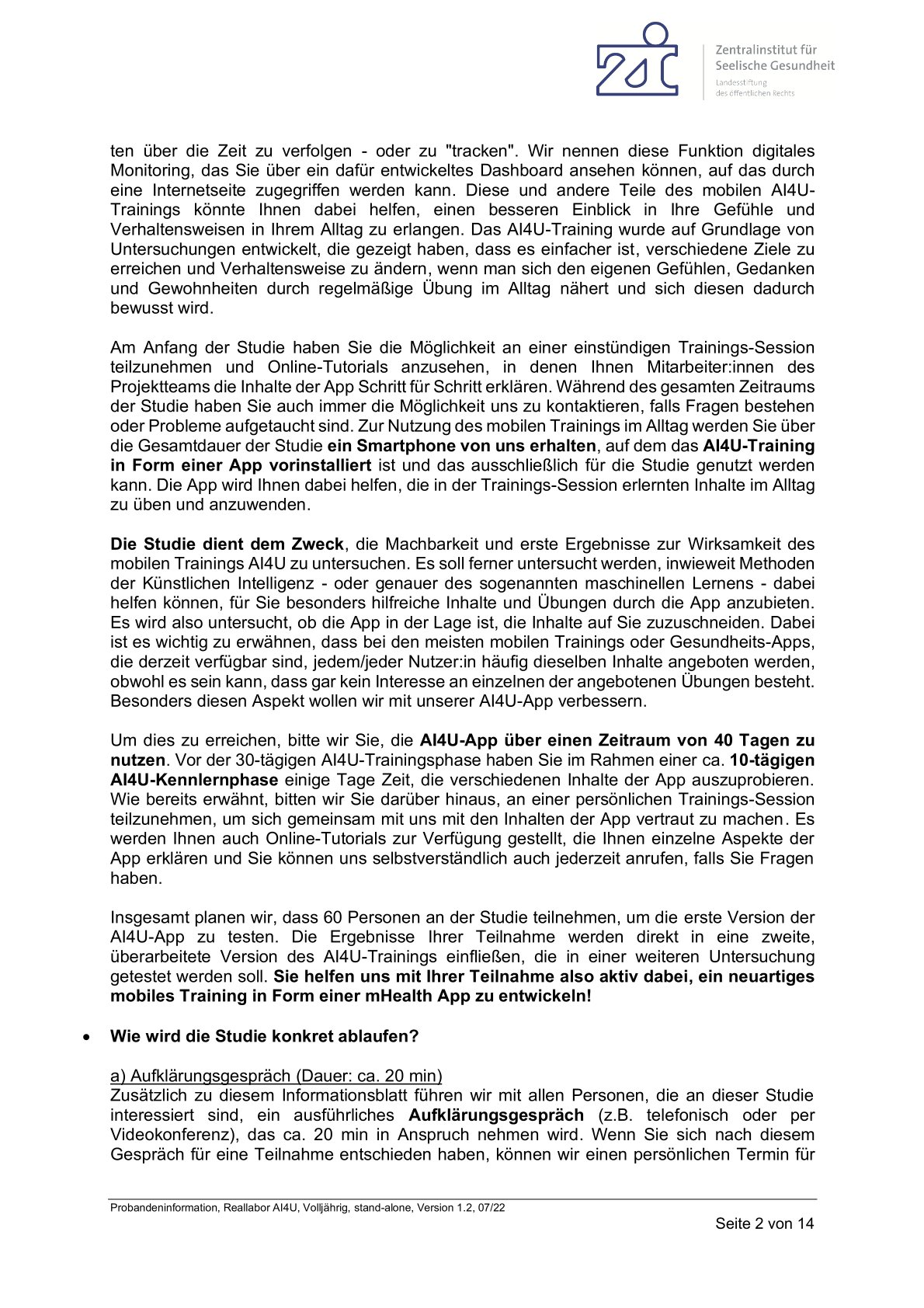

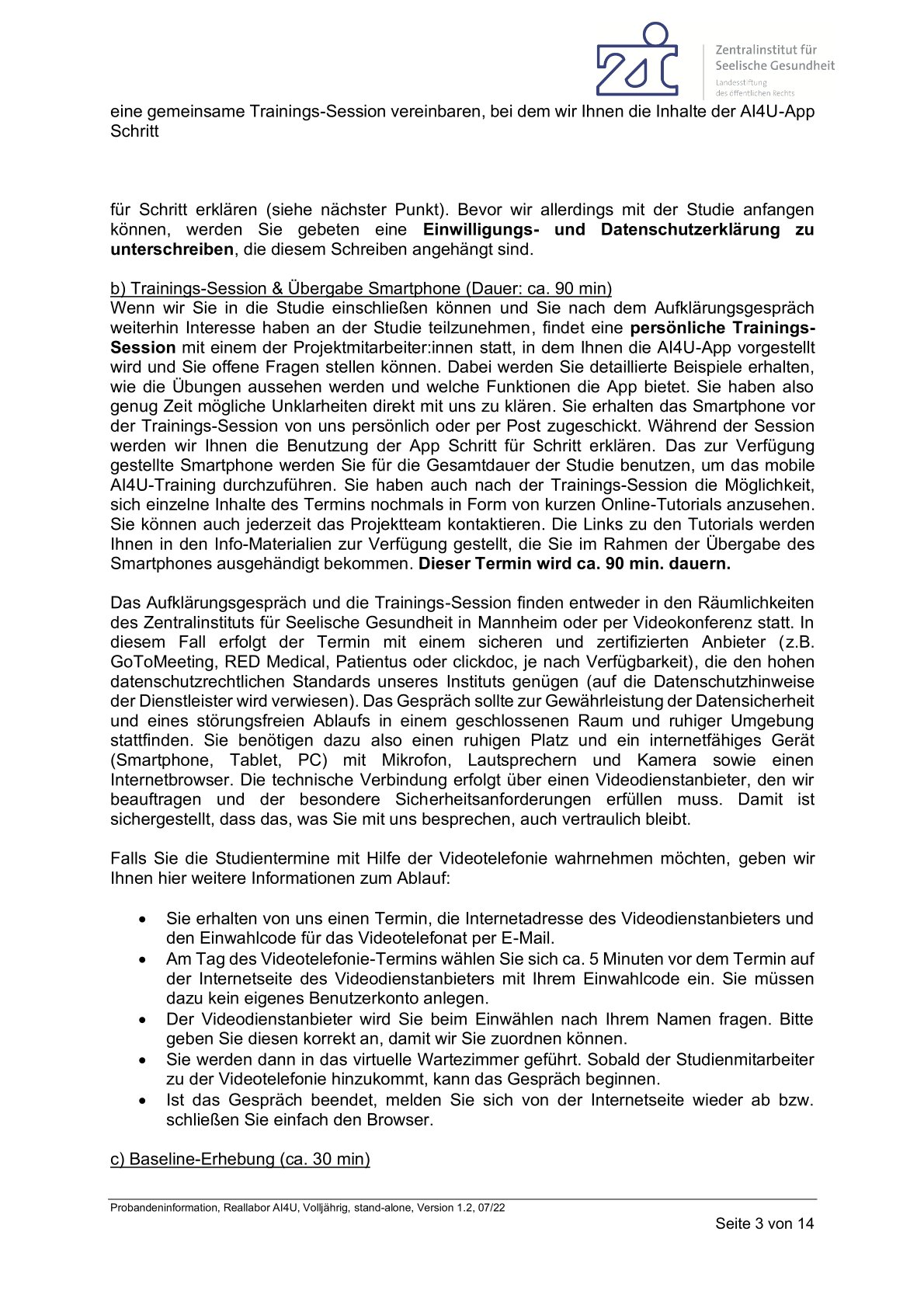

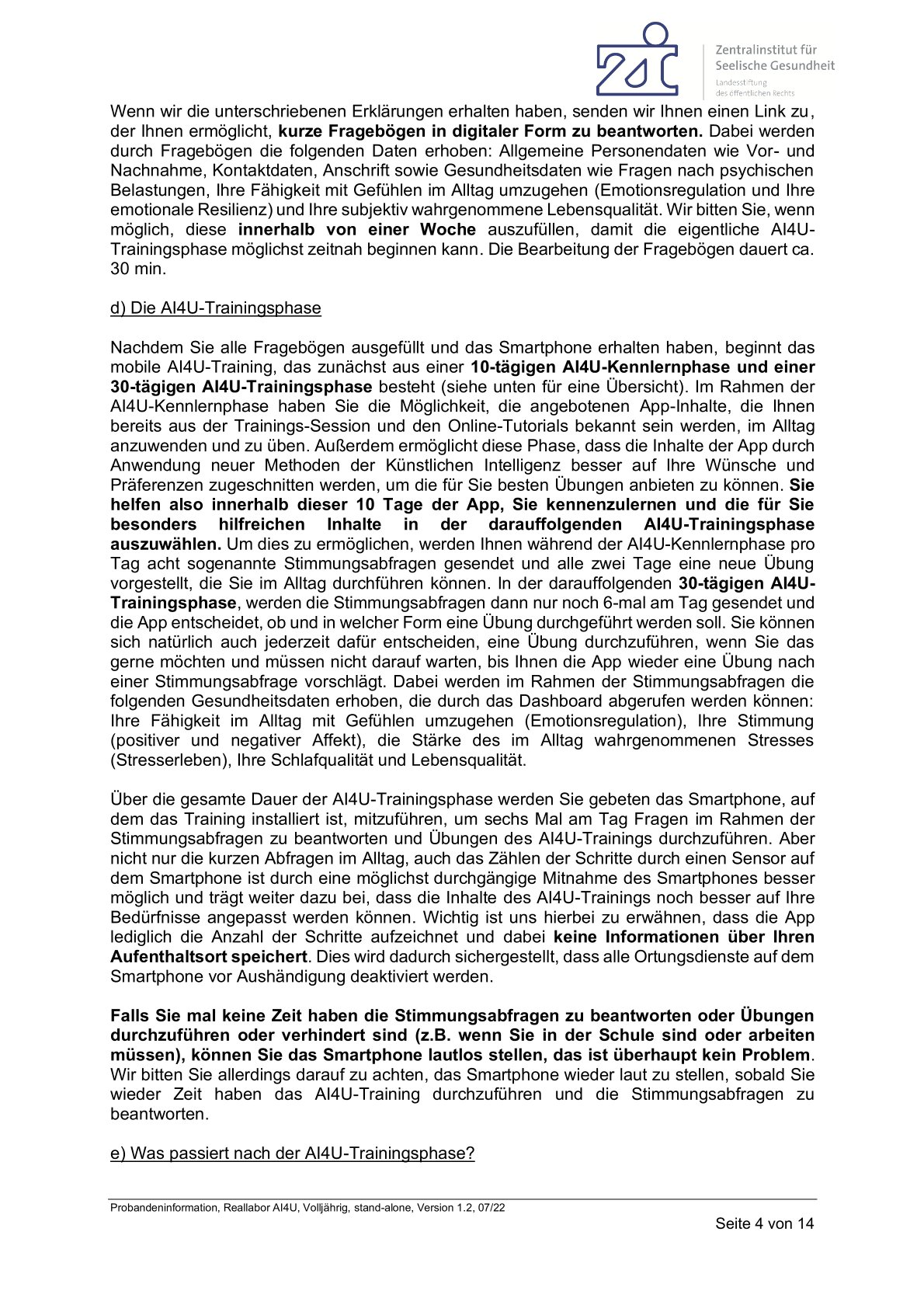

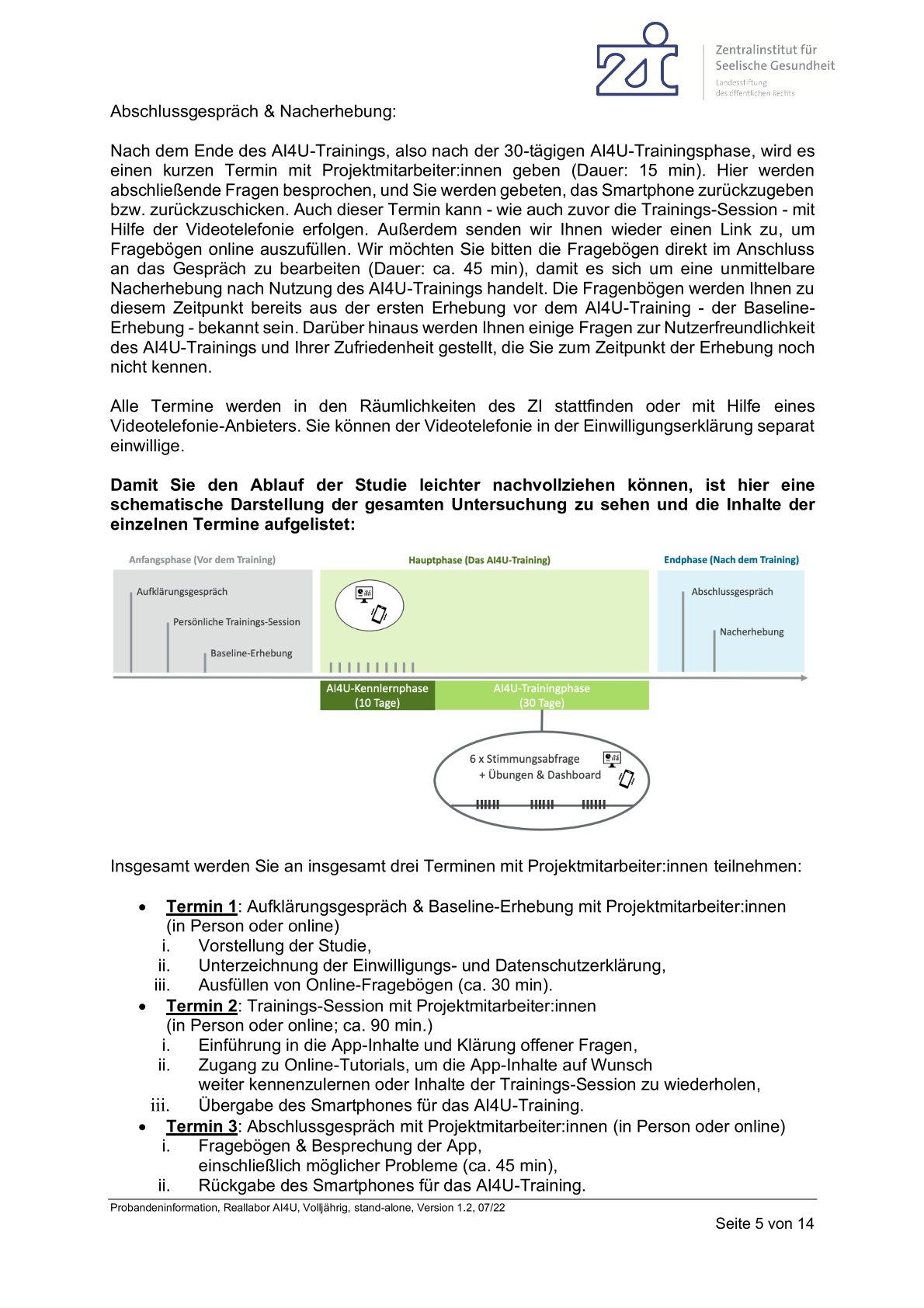

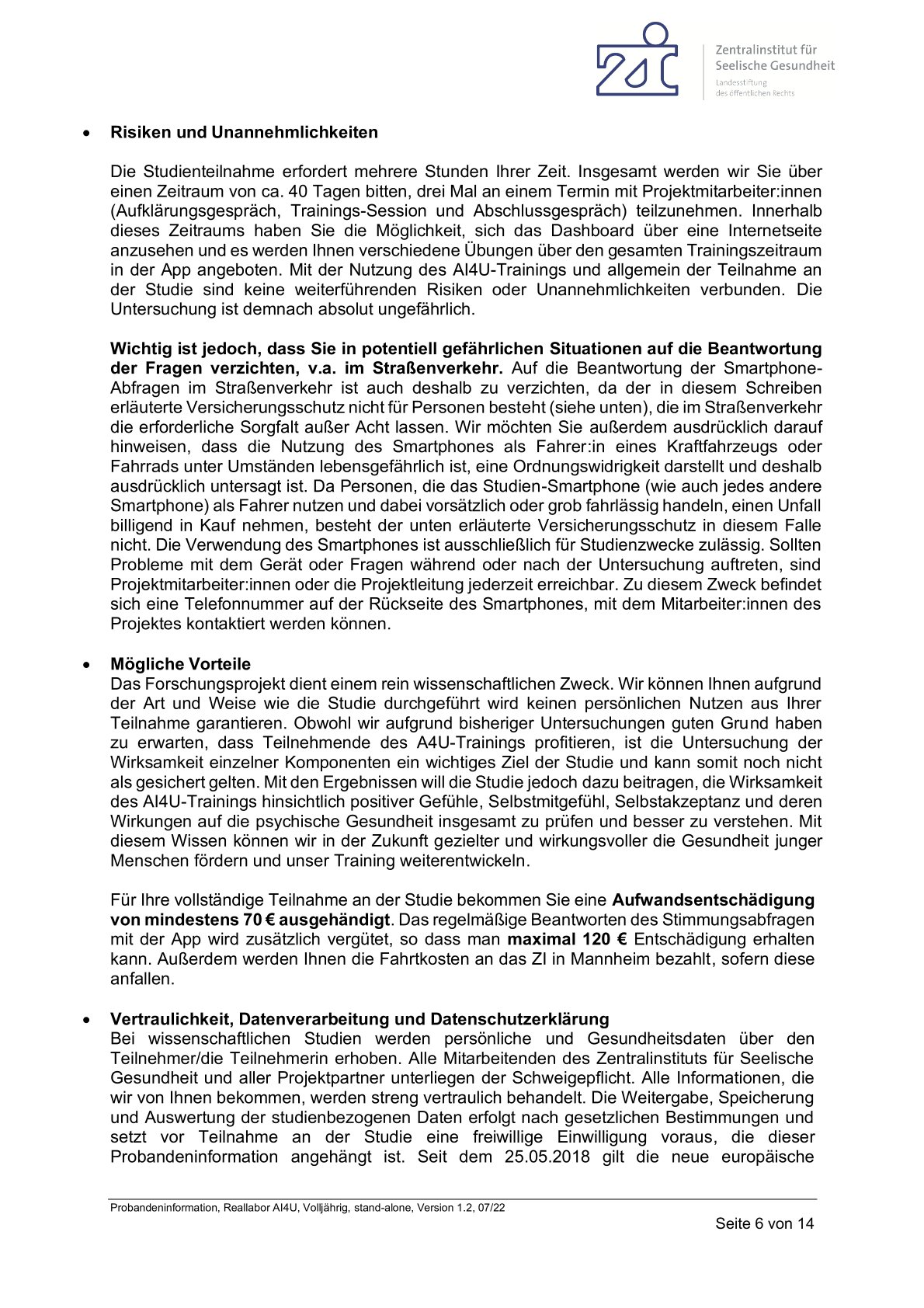

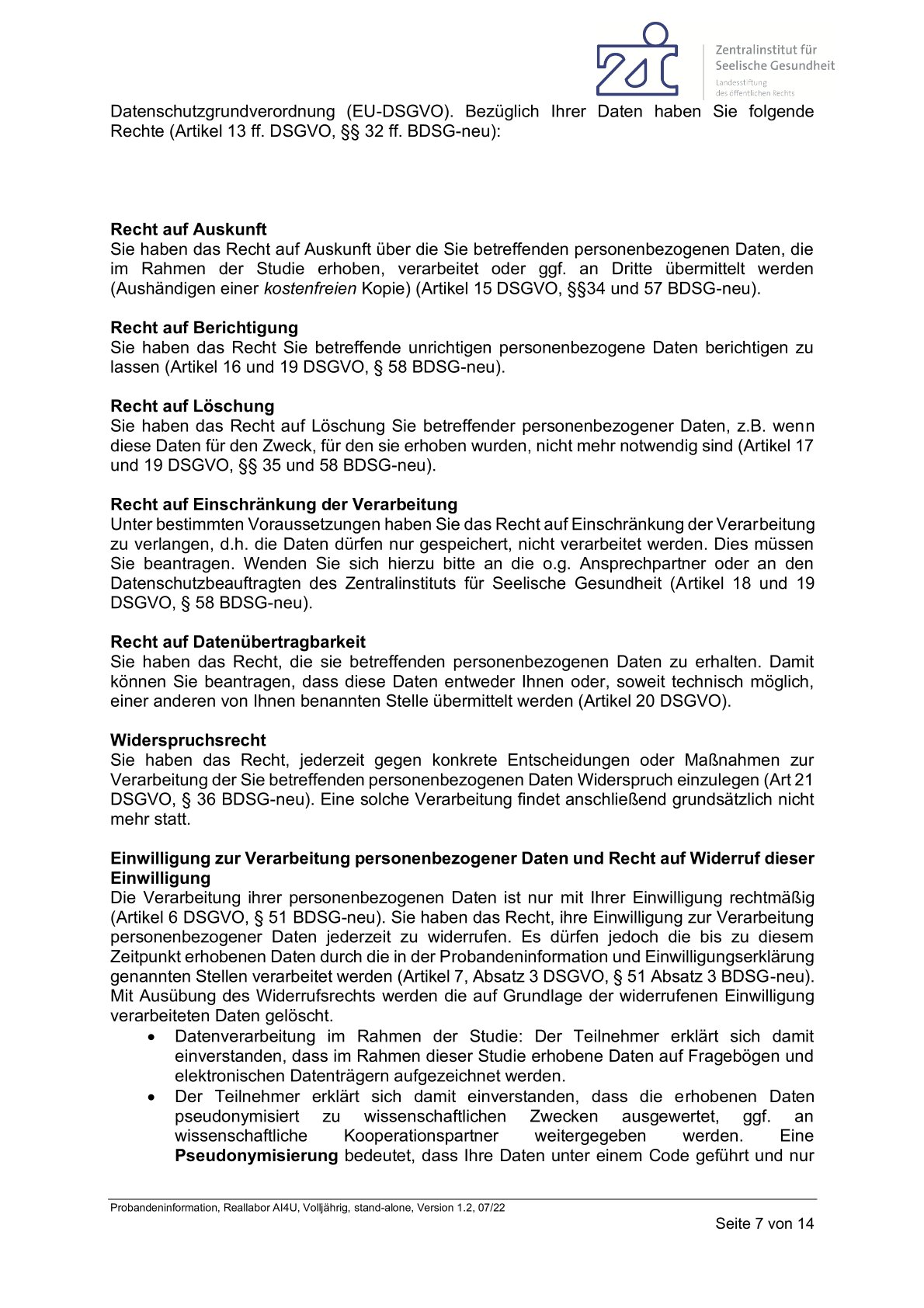

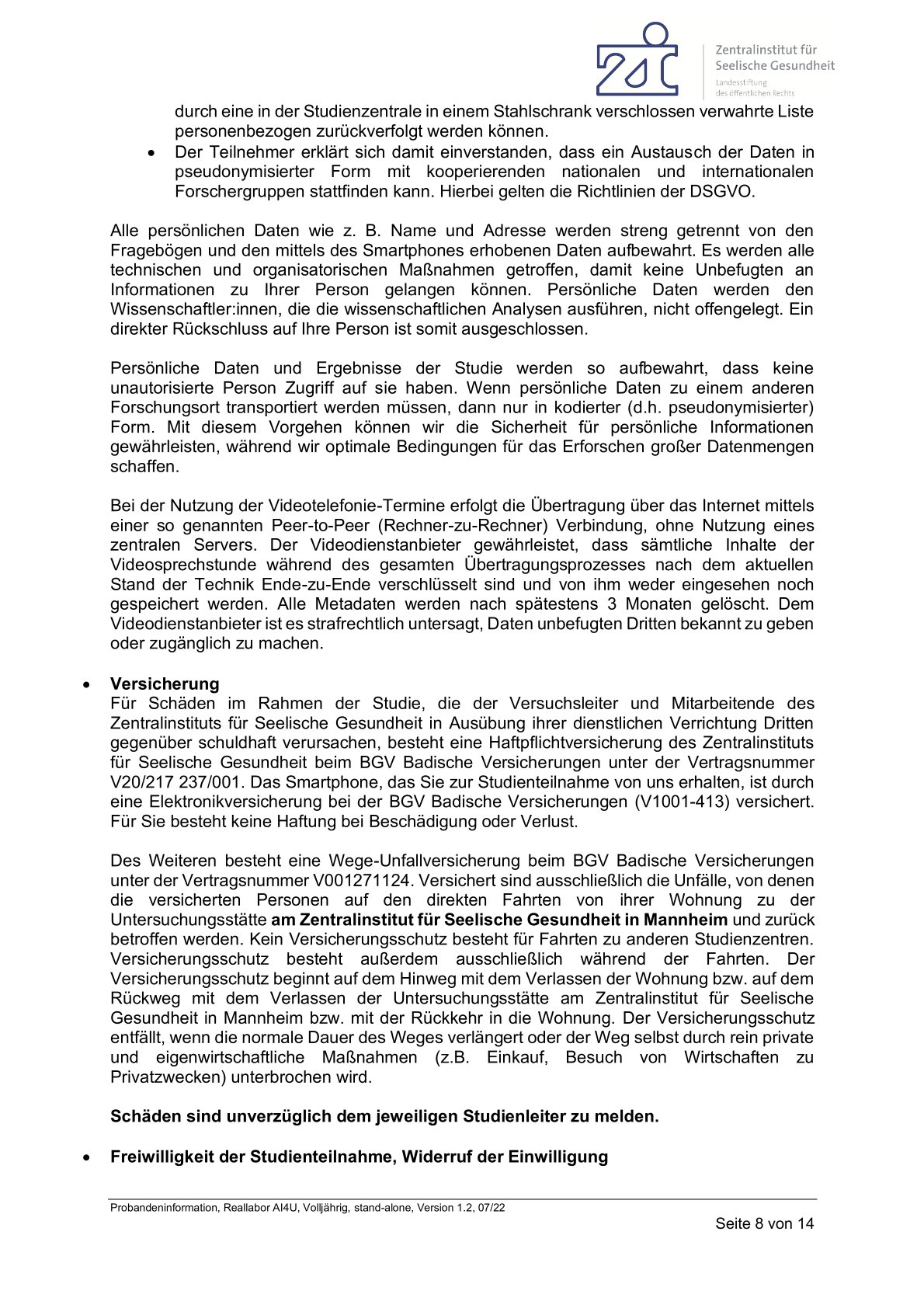

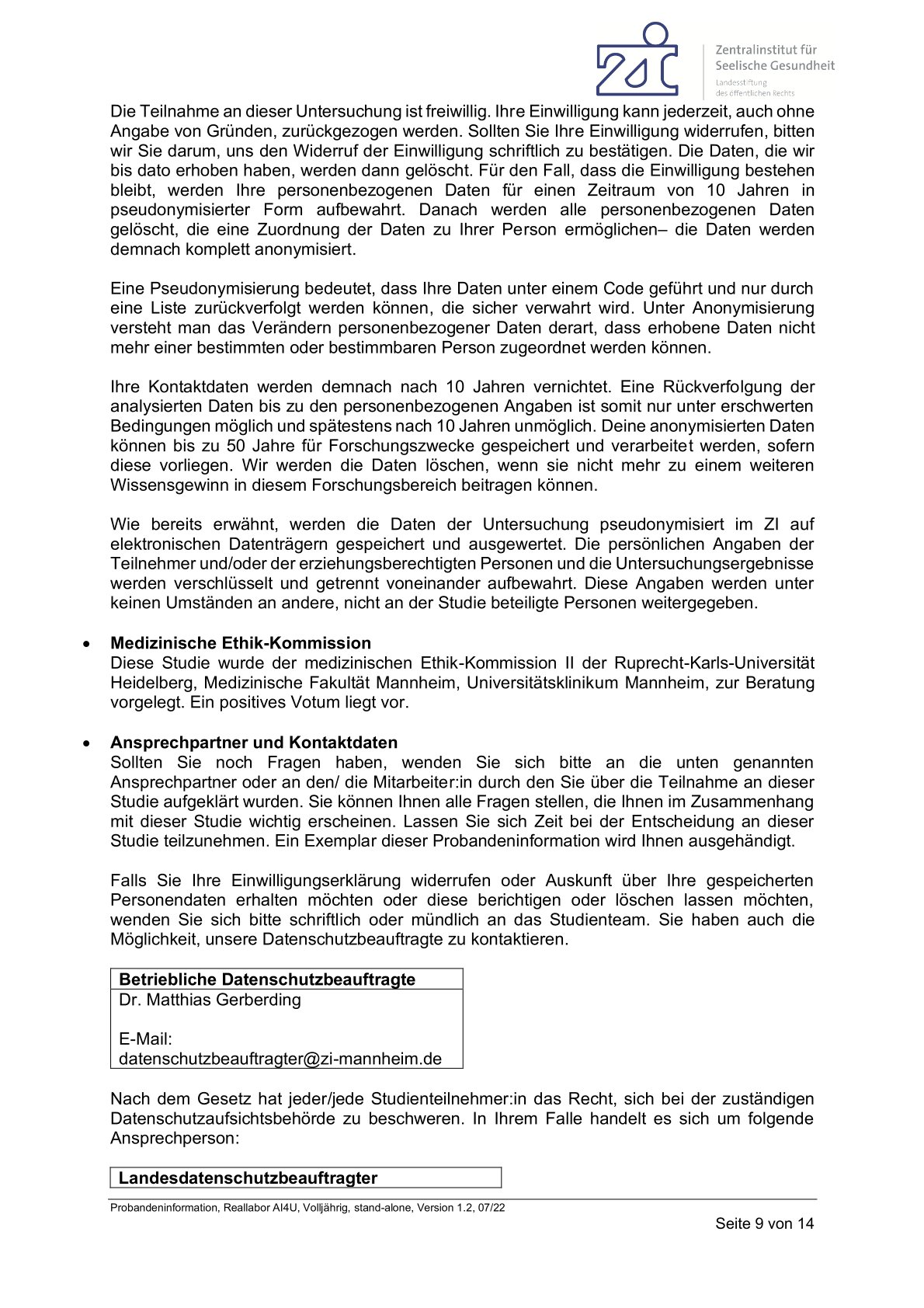

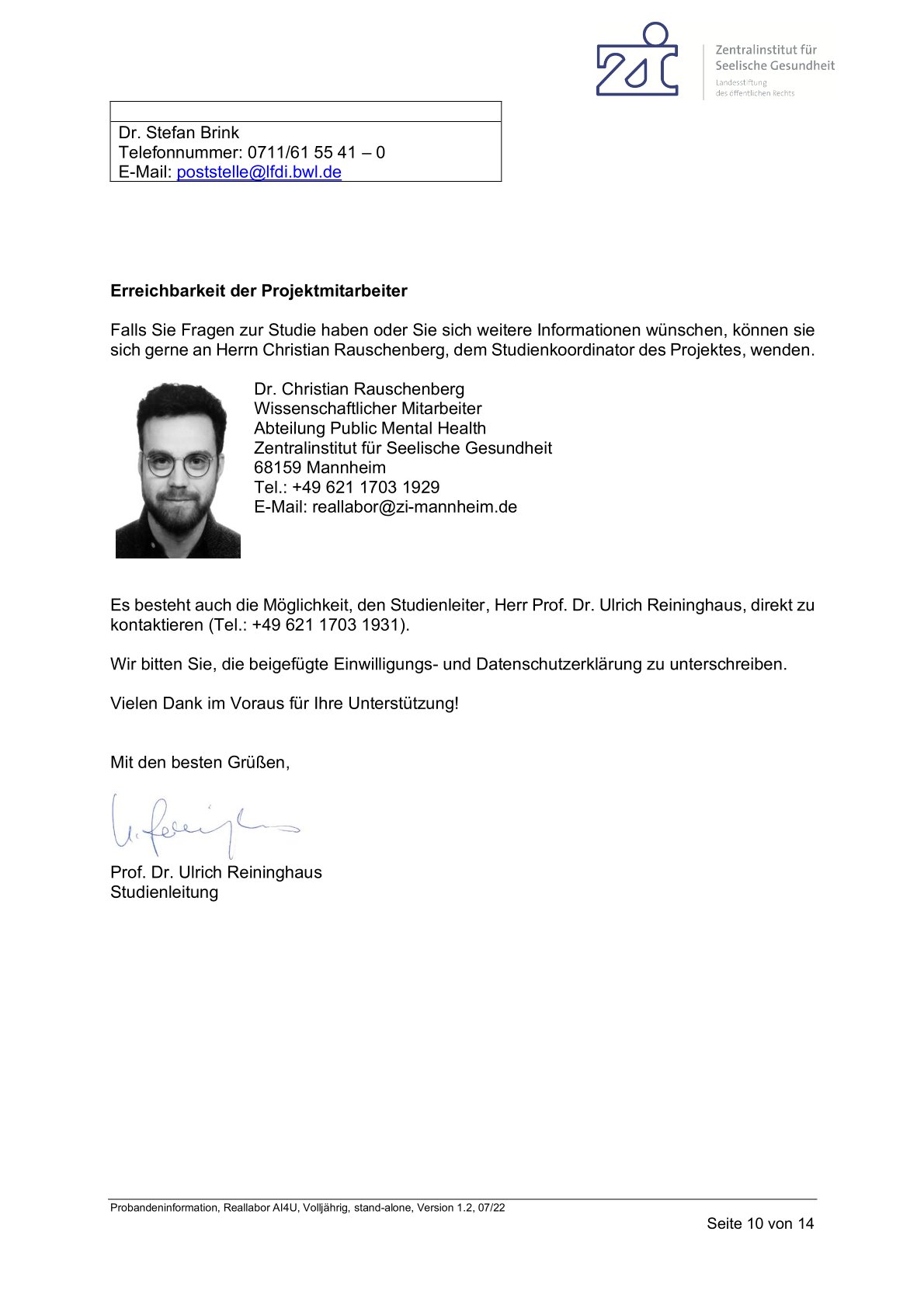

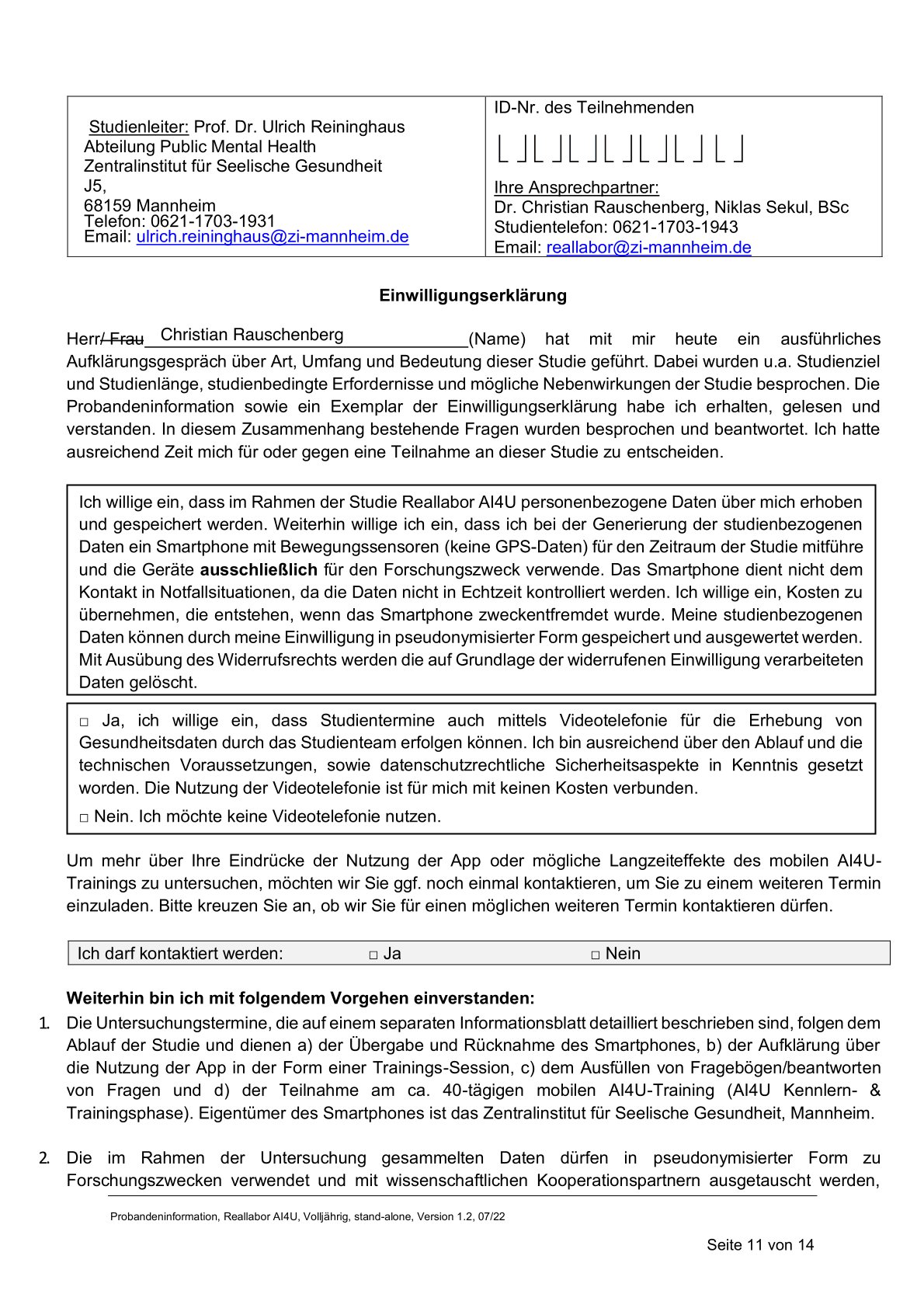

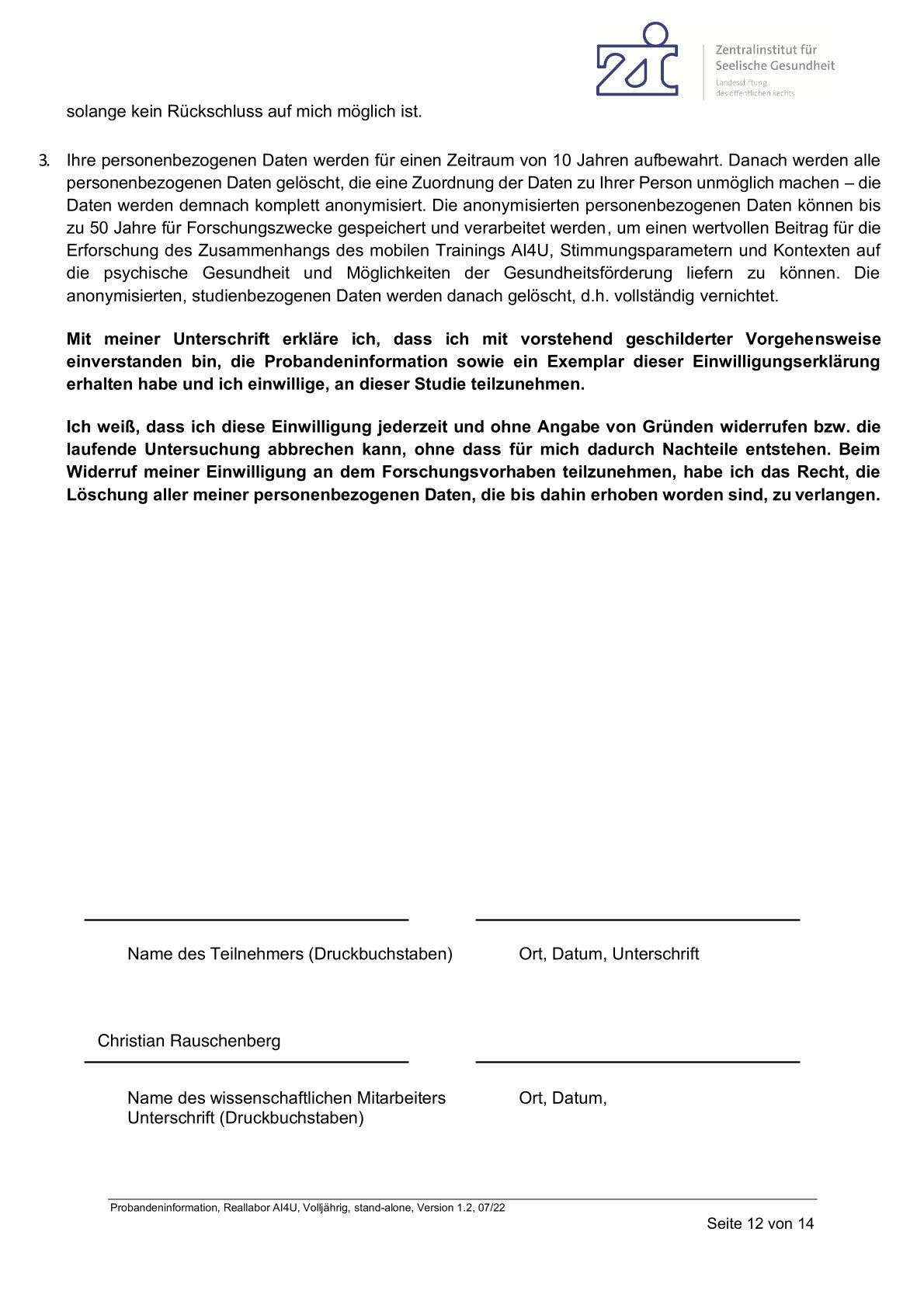

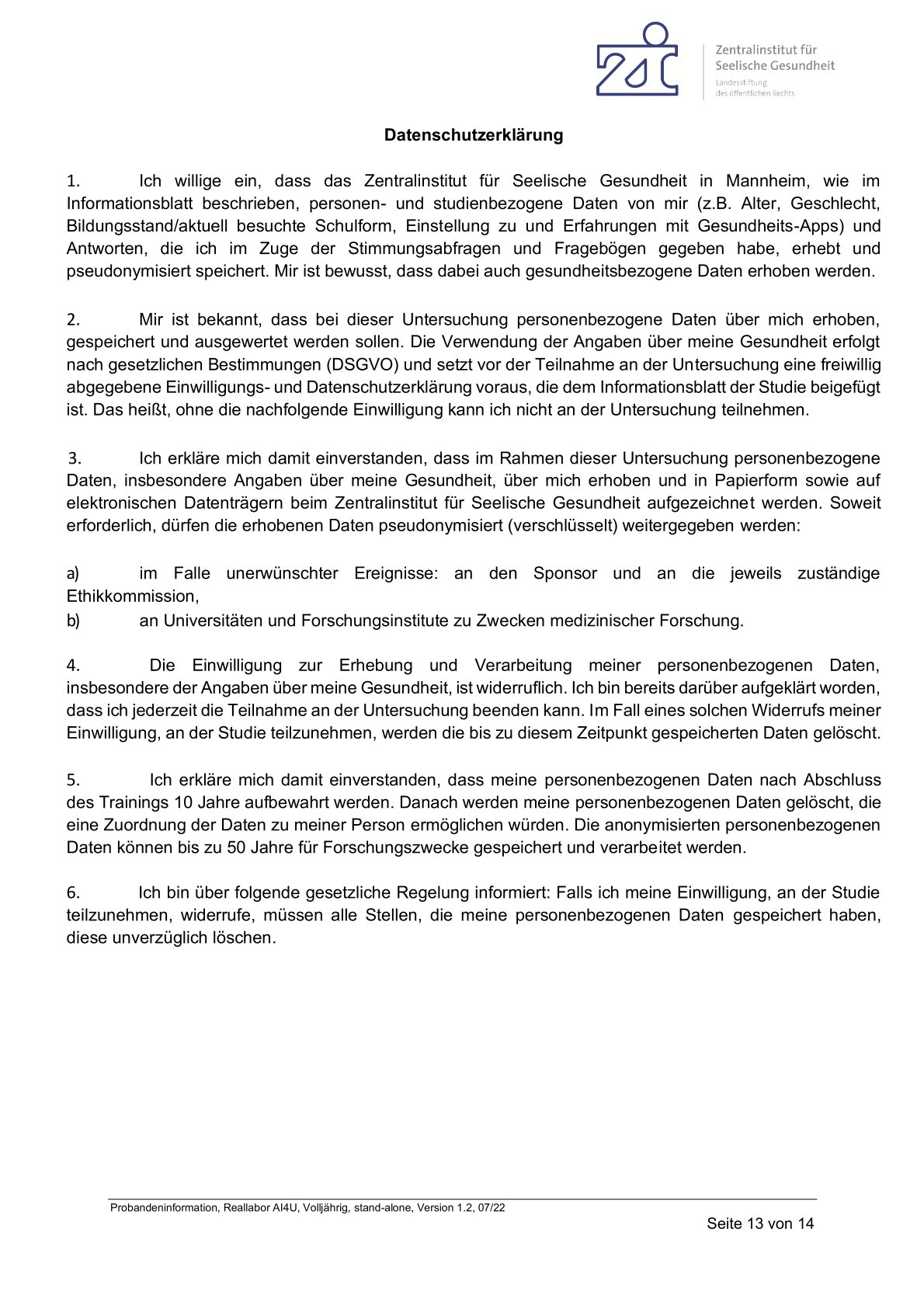

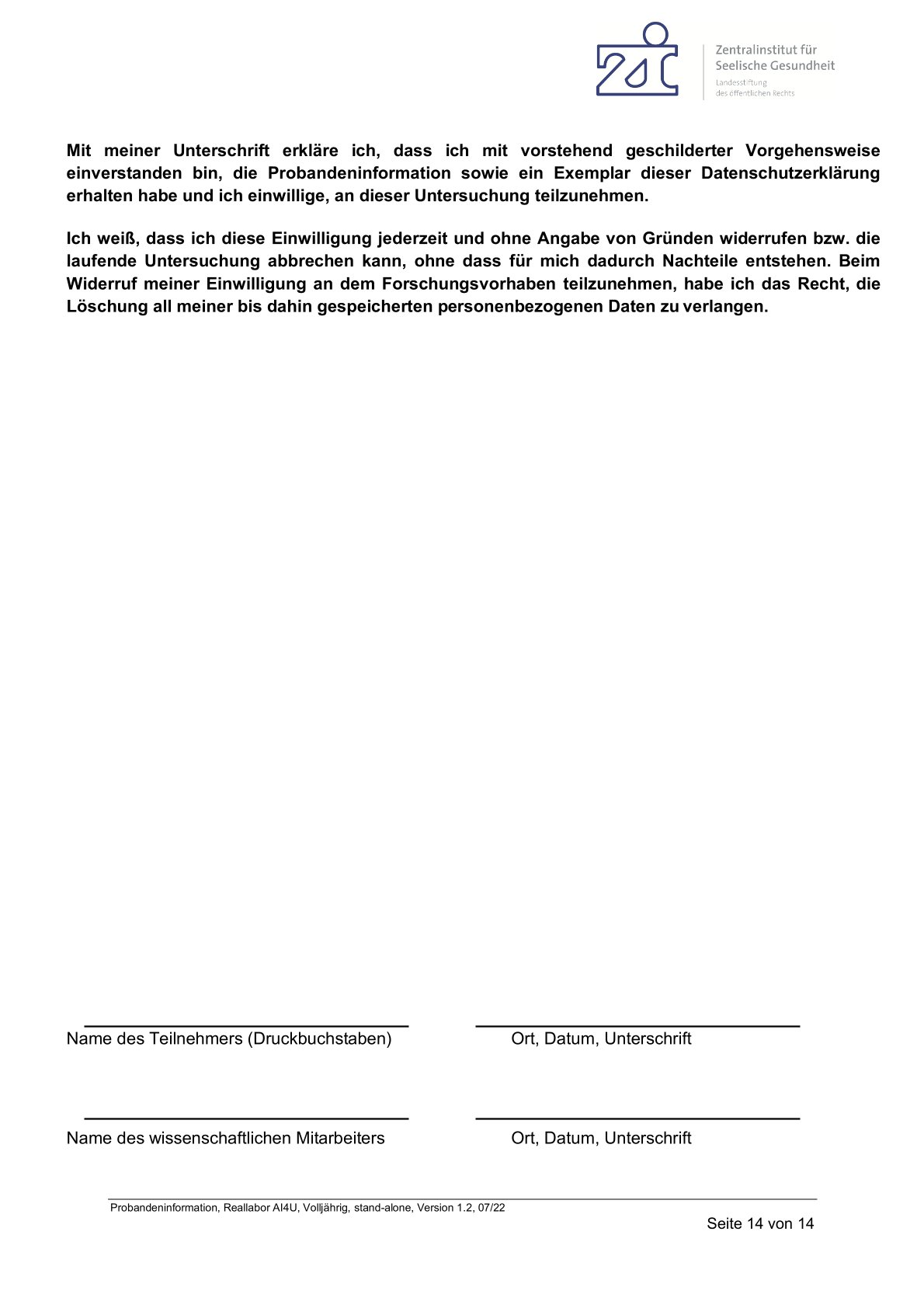

Supplement: Multimedia Appendix 3 [file jmir-v28-e85552-s003.docx]
