# Supplementary material for: Promoting Psychological Resilience and Well-Being in Youth With a Smartphone-Based Ecological Momentary mHealth Intervention: Secondary Analysis of a Microrandomized Trial
Source: J Med Internet Res. 2026 Jun 18;28:e85552. doi: 10.2196/85552 (PMC13280375; doi:10.2196/85552)
Supplement: Multimedia Appendix 4 [file jmir-v28-e85552-s004.docx]

Using generalized linear mixed models, we assessed whether the likelihood that an EMA prompt was not completed was associated with the time of day. Time of day was operationalized as hours, ie, as a continuous numeric variable from 0 to 24. Later time of day had no relevant effect on the likelihood of non-completion of an EMA prompt (OR=0.99, CI=0.99 to 1.00).

Similarly, we estimated whether the likelihood that an EMA prompt was not completed was associated with the number of the day since the participant started the training phase, with 0 indicating the first day of the training phase. A later training day had a small effect on the likelihood of non-completion of an EMA prompt as the confidence interval for the odds ratio did not include 1 (OR=1.03, CI=1.02 to 1.03).
