# Supplementary material for: Promoting Psychological Resilience and Well-Being in Youth With a Smartphone-Based Ecological Momentary mHealth Intervention: Secondary Analysis of a Microrandomized Trial
Source: J Med Internet Res. 2026 Jun 18;28:e85552. doi: 10.2196/85552 (PMC13280375; doi:10.2196/85552)
Supplement: Multimedia Appendix 5 [file jmir-v28-e85552-s005.docx]

**Table S1.** Characteristics of the 30-day training phase (number of EMA prompts and mean days of assessment, number and mean duration of EMI components).

| Characteristic |  |
| --- | --- |
| Completed EMA, n | 13059 |
| Days of assessment, mean (SD)^a^ | 24.51 (6.39) |
| **Initiated EMI, n** | 6667 |
| Compass of emotions | 791 |
| Counting your breath | 847 |
| My calm and safe place | 858 |
| Breathing with breaks | 837 |
| My compassionate companion | 844 |
| Emotion as a wave | 778 |
| Journal of joyful moments | 856 |
| Positive data log | 856 |
| **Duration of EMI in sec, mean (SD)^b^** | 126.39 (728.02) |
| Compass of emotions | 92.78 (136.27) |
| Counting your breath | 155.64 (149.18) |
| My calm and safe place ^b^ | 216.14 (1994.32) |
| Breathing with breaks | 157.52 (151.66) |
| My compassionate companion | 149.13 (174.74) |
| Emotion as a wave | 156.58 (162.83) |
| Journal of joyful moments | 41.76 (66.99) |
| Positive data log | 42.81 (41.38) |

**^a^Days of assessment: days with at least one completed EMA prompt; ^b^ One outlier with a duration of 262.41 hours for the completion of the EMI component was removed from the data set for this calculation**
