# Supplementary material for: Promoting Psychological Resilience and Well-Being in Youth With a Smartphone-Based Ecological Momentary mHealth Intervention: Secondary Analysis of a Microrandomized Trial
Source: J Med Internet Res. 2026 Jun 18;28:e85552. doi: 10.2196/85552 (PMC13280375; doi:10.2196/85552)
Supplement: Multimedia Appendix 6 [file jmir-v28-e85552-s006.docx]

**Table S1.** Spearman’s rank correlations of the person means of momentary positive affect, momentary negative affect, momentary stress, and momentary resilience with psychological distress at baseline^a^ and resilience at baseline^b^.

|  | Psychological distress at baseline | | Resilience at baseline | |
| --- | --- | --- | --- | --- |
|  | ρ | *P* | ρ | *P* |
| Positive affect | -0.47 | <.001 | 0.40 | <.001 |
| Negative affect | 0.43 | <.001 | -0.26 | <.001 |
| Stress | 0.28 | <.001 | -0.17 | .03 |
| Resilience | -0.44 | <.001 | 0.52 | <.001 |

^a^Psychological distress at baseline was measured using the Kessler Psychological Distress Scale [1].

^b^Resilience at baseline was measured using the Connor-Davidson Resilience Scale [2].

# References

1. Kessler RC, Andrews G, Colpe LJ, Hiripi E, Mroczek DK, Normand SL, et al. Short screening scales to monitor population prevalences and trends in non-specific psychological distress. Psychol Med. 2002 Aug;32(6):959-76. PMID: 12214795. doi: 10.1017/s0033291702006074.

2. Connor KM, Davidson JR. Development of a new resilience scale: the Connor-Davidson Resilience Scale (CD-RISC). Depress Anxiety. 2003;18(2):76-82. PMID: 12964174. doi: 10.1002/da.10113.
