# Supplementary material for: Promoting Psychological Resilience and Well-Being in Youth With a Smartphone-Based Ecological Momentary mHealth Intervention: Secondary Analysis of a Microrandomized Trial
Source: J Med Internet Res. 2026 Jun 18;28:e85552. doi: 10.2196/85552 (PMC13280375; doi:10.2196/85552)
Supplement: Multimedia Appendix 7 [file jmir-v28-e85552-s007.docx]

**Table S1.** Sensitivity analysis for type of EMI component: Regression coefficients, 95% confidence intervals, *P*-values and effect sizes of linear mixed model for effects of initiating a specific type of EMI component at t_n-1_ on well-being at t_n_ (hypothesis 1)^a^.

| Outcome: Positive affect at t_n_ | | | | | | | |
| --- | --- | --- | --- | --- | --- | --- | --- |
|  | *b* | | 95% CI | *P* | | *d* | |
| Breathing Exercise at t_n-1_ | -0.02 | | -0.08 - 0.04 | .49 | | -0.02 | |
| Compass of Emotions at t_n-1_ | 0.00 | | -0.08 - 0.08 | .93 | | 0.00 | |
| Positive Imagery at t_n-1_ | 0.01 | | -0.04 - 0.06 | .62 | | 0.01 | |
| Positive Refocusing at t_n-1_ | 0.00 | | -0.05 - 0.06 | .89 | | 0.01 | |
| Positive affect at t_n-1_ | 0.44 | | 0.42 - 0.46 | <.001 | | 0.50 | |
| Outcome: Negative affect at t_n_ | | | | | | | |
|  | *b* | | 95% CI | *P* | | *d* | |
| Breathing Exercise at t_n-1_ | -0.02 | | -0.08 - 0.04 | .51 | | -0.02 | |
| Compass of Emotions at t_n-1_ | -0.02 | | -0.1 - 0.06 | .68 | | -0.02 | |
| Positive Imagery at t_n-1_ | -0.03 | | -0.08 - 0.02 | .24 | | -0.03 | |
| Positive Refocusing at t_n-1_ | -0.03 | | -0.09 - 0.03 | .29 | | -0.04 | |
| Negative affect at t_n-1_ | 0.41 | | 0.39 - 0.43 | <.001 | | 0.46 | |
| Outcome: Stress at t_n_ | | | | | | | |
|  | *b* | 95% CI | | | *P* | | *d* |
| Breathing Exercise at t_n-1_ | 0.01 | | -0.05 - 0.07 | .65 | | 0.02 | |
| Compass of Emotions at t_n-1_ | -0.01 | | -0.09 - 0.07 | .82 | | -0.01 | |
| Positive Imagery at t_n-1_ | 0.00 | | -0.05 - 0.05 | .98 | | 0.00 | |
| Positive Refocusing at t_n-1_ | 0.03 | | -0.03 - 0.09 | .30 | | 0.04 | |
| Stress at t_n-1_ | 0.39 | | 0.37 - 0.41 | <.001 | | 0.42 | |

^a^Adjusted for potential confounding by age, gender, allocation of EMI component, MRT, and psychological distress at baseline.

**Table S2.** Sensitivity analysis for type of EMI component: Regression coefficients, 95% confidence intervals, *P*-values and effect sizes of linear mixed model for effects of initiating a specific type of EMI component at t_n-1_ and well-being at t_n_ by well-being at t_n-1_ (hypothesis 2)^a^.

| Outcome: Positive affect at t_n_ | | | | |
| --- | --- | --- | --- | --- |
|  | *b* | 95% CI | *P* | *d* |
| Breathing Exercise at t_n-1_ | -0.02 | -0.08 - 0.04 | .50 | -0.02 |
| Compass of Emotions at t_n-1_ | 0.00 | -0.08 - 0.08 | .92 | 0.00 |
| Positive Imagery at t_n-1_ | 0.02 | -0.04 - 0.07 | .56 | 0.02 |
| Positive Refocusing at t_n-1_ | 0.01 | -0.05 - 0.06 | .85 | 0.02 |
| Positive affect at t_n-1_ | 0.42 | 0.40 - 0.45 | <.001 | 0.48 |
| Interaction Breathing Exercise at t_n-1_ × Positive affect at t_n-1_ | -0.03 | -0.09 - 0.03 | .27 | -0.04 |
| Interaction Compass of Emotions at t_n-1_ × Positive affect at t_n-1_ | 0.04 | -0.05 - 0.12 | .40 | 0.04 |
| Interaction Positive Imagery at t_n-1_ × Positive affect at t_n-1_ | 0.09 | 0.04 - 0.14 | <.001 | 0.10 |
| High positive affect | 0.11 | 0.02 – 0.19 | .01 | 0.12 |
| Low positive affect | -0.08 | -0.16 – 0.01 | .09 | -0.09 |
| High vs low positive affect | 0.18 | 0.08 – 0.29 | <.001 | 0.21 |
| Interaction Positive Refocusing at t_n-1_ × Positive affect at t_n-1_ | 0.05 | -0.01 - 0.11 | .11 | 0.06 |
| Outcome: Negative affect at t_n_ | | | | |
|  | *b* | 95% CI | *P* | *d* |
| Breathing Exercise at t_n-1_ | -0.02 | -0.08 - 0.04 | .55 | -0.02 |
| Compass of Emotions at t_n-1_ | -0.02 | -0.10 - 0.06 | .68 | -0.02 |
| Positive Imagery at t_n-1_ | -0.03 | -0.08 - 0.02 | .23 | -0.04 |
| Positive Refocusing at t_n-1_ | -0.03 | -0.09 - 0.03 | .27 | -0.04 |
| Negative affect at t_n-1_ | 0.43 | 0.41 - 0.46 | <.001 | 0.49 |
| Interaction Breathing Exercise at t_n-1_× Negative affect at t_n-1_ | -0.08 | -0.14 - -0.02 | .01 | -0.09 |
| High negative affect | -0.10 | -0.19 - -0.00 | .04 | -0.11 |
| Low negative affect | 0.06 | -0.03 – 0.16 | .28 | 0.07 |
| High vs low negative affect | -0.16 | -0.28 - -0.05 | .01 | -0.18 |
| Interaction Compass of Emotions at t_n-1_ × Negative affect at t_n-1_ | -0.04 | -0.12 - 0.04 | .37 | -0.04 |
| Interaction Positive Imagery at t_n-1_ × Negative affect at t_n-1_ | -0.01 | -0.07 - 0.04 | .57 | -0.02 |
| Interaction Positive Refocusing at t_n-1_× Negative affect at t_n-1_ | 0.06 | -0.12 - 0.00 | .06 | -0.07 |
| Outcome: Stress at t_n_ | | | | |
|  | *b* | 95% CI | *P* | *d* |
| Breathing Exercise at t_n-1_ | 0.01 | -0.05 - 0.07 | .65 | 0.02 |
| Compass of Emotions at t_n-1_ | -0.01 | -0.09 - 0.08 | .87 | -0.01 |
| Positive Imagery at t_n-1_ | 0.00 | -0.05 - 0.05 | .96 | 0.00 |
| Positive Refocusing at t_n-1_ | 0.03 | -0.03 - 0.09 | .31 | 0.03 |
| Stress at t_n-1_ | 0.367 | 0.34 - 0.39 | <.001 | 0.40 |
| Interaction Breathing Exercise at t_n-1_× Stress at t_n-1_ | -0.02 | -0.08 - 0.04 | .56 | -0.02 |
| Interaction Compass of Emotions at t_n-1_× Stress at t_n-1_ | 0.11 | 0.02 - 0.19 | .02 | 0.12 |
| High stress | 0.10 | -0.04 - 0.24 | .23 | 0.11 |
| Low stress | -0.11 | -0.25 - 0.03 | .13 | -0.15 |
| High vs low stress | 0.21 | 0.04 - 0.39 | .02 | 0.23 |
| Interaction Positive Imagery at t_n-1_× Stress at t_n-1_ | 0.03 | -0.02 - 0.08 | .28 | 0.03 |
| Interaction Positive Refocusing at t_n-1_× Stress at t_n-1_ | 0.10 | 0.04 - 0.17 | <.01 | 0.11 |
| High stress | 0.13 | 0.03 – 0.23 | <.01 | 0.15 |
| Low stress | -0.07 | -0.18 – 0.03 | .21 | -0.08 |
| High vs low Stress | 0.21 | 0.08 – 0.33 | <.01 | 0.23 |

^a^Adjusted for potential confounding by age, gender, allocation of EMI component, MRT, and psychological distress at baseline.

**Table S3.** Sensitivity analysis for type of EMI component: Regression coefficients, 95% confidence intervals, *P*-values and effect sizes of linear mixed model for effect of initiation of a specific type of EMI component at t_n-1_ on change in resilience from t_n-1_ to t_n_ : mediator model (hypothesis 3)^a^.

| Outcome: Change in resilience | | | | |
| --- | --- | --- | --- | --- |
|  | *b* | 95% CI | *P* | *d* |
| Breathing Exercise at t_n-1_ |  |  |  |  |
| Controlled for positive affect at t_n-1_ | 0.03 | -0.04 - 0.11 | .41 | 0.03 |
| Controlled for negative affect at t_n-1_ | 0.02 | -0.06 - 0.09 | .64 | 0.02 |
| Controlled for stress affect at t_n-1_ | 0.03 | -0.05 - 0.10 | .48 | 0.02 |
| Compass of Emotions at t_n-1_ |  |  |  |  |
| Controlled for positive affect at t_n-1_ | 0.04 | -0.07 - 0.14 | .49 | 0.03 |
| Controlled for negative affect at t_n-1_ | 0.03 | -0.08 - 0.13 | .60 | 0.02 |
| Controlled for stress affect at t_n-1_ | 0.04 | -0.07 - 0.15 | .46 | 0.03 |
| Positive Imagery at t_n-1_ |  |  |  |  |
| Controlled for positive affect at t_n-1_ | 0.01 | -0.05 - 0.08 | .69 | 0.01 |
| Controlled for negative affect at t_n-1_ | 0.01 | -0.06 - 0.07 | .80 | 0.01 |
| Controlled for stress affect at t_n-1_ | 0.02 | -0.05 - 0.08 | .65 | 0.01 |
| Positive Refocusing at t_n-1_ |  |  |  |  |
| Controlled for positive affect at t_n-1_ | 0.02 | -0.05 - 0.10 | .59 | 0.02 |
| Controlled for negative affect at t_n-1_ | 0.03 | -0.05 - 0.10 | .59 | 0.02 |
| Controlled for stress affect at t_n-1_ | 0.02 | -0.05 - 0.10 | .59 | 0.02 |

^a^Adjusted for potential confounding by age, gender, allocation of EMI component, MRT, and psychological distress at baseline.

**Table S4.** Sensitivity analysis for type of EMI component: Regression coefficients, 95% confidence intervals, *P*-values and effect sizes of linear mixed model for effects of initiating an EMI component at t_n-1_ on changes in well-being from t_n-1_ to t_n_ via changes in resilience from t_n-1_ to t_n_: (outcome model, hypothesis 3)^a^.

| Outcome: Change in positive affect | | | | |
| --- | --- | --- | --- | --- |
|  | *b* | 95% CI | *P* | *d* |
| Change in resilience | 0.22 | 0.21 - 0.24 | <.001 | 0.27 |
| Breathing Exercise at t_n-1_ | -0.03 | -0.08 - 0.03 | .37 | -0.03 |
| Compass of Emotions at t_n-1_ | 0.00 | -0.08 - 0.08 | .96 | 0.00 |
| Positive Imagery at t_n-1_ | 0.01 | -0.04 - 0.06 | .69 | 0.01 |
| Positive Refocusing at t_n-1_ | 0.00 | -0.06 - 0.06 | .99 | 0.00 |
| Outcome: Change in negative affect | | | | |
|  | *b* | 95% CI | *P* | *d* |
| Change in resilience | -0.16 | -0.18 - -0.15 | <.001 | -0.19 |
| Breathing Exercise at t_n-1_ | -0.02 | -0.04 - 0.04 | .51 | -0.02 |
| Compass of Emotions at t_n-1_ | -0.01 | -0.09 - 0.07 | .79 | -0.01 |
| Positive Imagery at t_n-1_ | -0.03 | -0.08 - 0.02 | .25 | -0.03 |
| Positive Refocusing at t_n-1_ | -0.03 | -0.09 - 0.03 | .30 | -0.03 |
| Outcome: Change in stress | | | | |
|  | *b* | 95% CI | *P* | *d* |
| Change in resilience | -0.11 | -0.13 - -0.10 | <.001 | -0.13 |
| Breathing Exercise at t_n-1_ | 0.02 | -0.04 - 0.08 | .56 | 0.02 |
| Compass of Emotions at t_n-1_ | -0.01 | -0.09 - 0.08 | .88 | -0.01 |
| Positive Imagery at t_n-1_ | 0.00 | -0.05 - 0.05 | .99 | 0.00 |
| Positive Refocusing at t_n-1_ | 0.03 | -0.03 - 0.09 | .28 | 0.04 |

^a^Adjusted for potential confounding by age, gender, allocation of EMI component, MRT, and psychological distress.

**Table S5.** Sensitivity analysis for type of EMI component: Regression coefficients, 95% confidence intervals, and *P*-values of mediation model for effects of initiating an EMI component at t_n-1_ on changes in well-being from t_n-1_ to t_n_ via changes in resilience from t_n-1_ to t_n_ ( hypothesis 3)^a^.

| Outcome: Change in positive affect | | | |
| --- | --- | --- | --- |
|  | *b* | 95% CI | *P* |
| Breathing Exercise vs No EMI |  |  |  |
| Total effect | -0.02 | -0.08 - 0.04 | .51 |
| Direct effect | -0.03 | -0.08 - 0.03 | .36 |
| Indirect effect | 0.01 | -0.01 - 0.02 | .44 |
| Proportion mediated | -0.09 | -4.65 - 5.62 | .79 |
| Compass of Emotions vs No EMI |  |  |  |
| Total effect | 0.00 | -0.08 - 0.08 | .93 |
| Direct effect | -0.01 | -0.08 - 0.08 | .91 |
| Indirect effect | 0.01 | -0.01 - 0.03 | .46 |
| Proportion mediated | 0.09 | -3.97 - 4.03 | .83 |
| Positive Imagery vs No EMI |  |  |  |
| Total effect | 0.01 | -0.04 - 0.06 | .67 |
| Direct effect | 0.01 | -0.04 - 0.06 | .76 |
| Indirect effect | 0.00 | -0.01 - 0.02 | .71 |
| Proportion mediated | 0.12 | -2.62 - 3.76 | .71 |
| Positive Refocusing vs No EMI |  |  |  |
| Total effect | 0.00 | -0.06 - 0.06 | .97 |
| Direct effect | 0.00 | -0.06 - 0.05 | .92 |
| Indirect effect | 0.00 | -0.01 - 0.02 | .69 |
| Proportion mediated | 0.07 | -3.77 – 3.59 | .84 |
| Outcome: Change in negative affect | | | |
|  | *b* | 95% CI | *P* |
| Breathing Exercise vs No EMI |  |  |  |
| Total effect | -0.02 | -0.08 - 0.04 | .49 |
| Direct effect | -0.02 | -0.07 - 0.04 | .51 |
| Indirect effect | 0.00 | -0.01 - 0.01 | .73 |
| Proportion mediated | 0.07 | -2.06 - 2.09 | .78 |
| Compass of Emotions vs No EMI |  |  |  |
| Total effect | -0.01 | -0.10 - 0.06 | .72 |
| Direct effect | -0.01 | -0.09 - 0.07 | .82 |
| Indirect effect | -0.01 | -0.02 - 0.01 | .54 |
| Proportion mediated | 0.09 | -2.77 - 2.57 | .75 |
| Positive Imagery vs No EMI |  |  |  |
| Total effect | -0.03 | -0.08 - 0.02 | .28 |
| Direct effect | -0.03 | -0.08 - 0.02 | .27 |
| Indirect effect | 0.00 | -0.01 - 0.01 | .84 |
| Proportion mediated | 0.04 | -1.69 – 1.23 | .83 |
| Positive Refocusing vs No EMI |  |  |  |
| Total effect | -0.03 | -0.09 - 0.03 | .30 |
| Direct effect | -0.03 | -0.09 - 0.03 | .32 |
| Indirect effect | 0.00 | -0.01 - 0.01 | .69 |
| Proportion mediated | 0.06 | -1.14 – 1.40 | .72 |
| Outcome: Change in stress | | | |
|  | *b* | 95% CI | *P* |
| Breathing Exercise vs No EMI |  |  |  |
| Total effect | 0.02 | -0.04 - 0.08 | .65 |
| Direct effect | 0.02 | -0.04 - 0.08 | .60 |
| Indirect effect | 0.00 | -0.01 - 0.01 | .58 |
| Proportion mediated | -0.02 | -1.59 - 1.84 | .89 |
| Compass of Emotions vs No EMI |  |  |  |
| Total effect | -0.01 | -0.09 - 0.07 | .80 |
| Direct effect | -0.01 | -0.09 - 0.08 | .86 |
| Indirect effect | 0.00 | -0.01 - 0.01 | .46 |
| Proportion mediated | 0.03 | -2.49 - 1.43 | .88 |
| Positive Imagery vs No EMI |  |  |  |
| Total effect | 0.00 | -0.06 - 0.05 | .97 |
| Direct effect | 0.00 | -0.05 - 0.05 | .99 |
| Indirect effect | 0.00 | -0.01 - 0.01 | .69 |
| Proportion mediated | 0.02 | -1.56 - 1.59 | .88 |
| Positive Refocusing vs No EMI |  |  |  |
| Total effect | 0.03 | -0.03 - 0.09 | .32 |
| Direct effect | 0.03 | -0.03 - 0.09 | .30 |
| Indirect effect | 0.00 | -0.01 - 0.01 | .72 |
| Proportion mediated | -0.02 | -1.31 – 0.81 | .83 |

^a^Adjusted for potential confounding by age, gender, allocation of EMI component, MRT, and psychological distress at baseline.
