# Supplementary material for: Promoting Psychological Resilience and Well-Being in Youth With a Smartphone-Based Ecological Momentary mHealth Intervention: Secondary Analysis of a Microrandomized Trial
Source: J Med Internet Res. 2026 Jun 18;28:e85552. doi: 10.2196/85552 (PMC13280375; doi:10.2196/85552)
Supplement: Multimedia Appendix 8 [file jmir-v28-e85552-s008.docx]

**Table S1.** Sensitivity analysis for reason of EMI component non-initiation: Regression coefficients, 95% confidence intervals, *P*-values and effect sizes of linear mixed model for effects of actively accepting/ refusing a suggested EMI component vs no EMI component being suggested at t_n-1_ on well-being at t_n_ (hypothesis 1)^a^.

| Outcome: Positive affect at t_n_ | | | | | | |
| --- | --- | --- | --- | --- | --- | --- |
|  | *b* | | 95% CI | *P* | d | |
| Accept EMI component at t_n-1_ | 0.00 | | -0.06 - 0.06 | .97 | 0.00 | |
| Refuse EMI component at t_n-1_ | 0.01 | | -0.06 - 0.08 | .71 | 0.01 | |
| Positive affect at t_n-1_ | 0.45 | | 0.43 - 0.47 | <.001 | 0.51 | |
| Outcome: Negative affect at t_n_ | | | | | | |
|  | *b* | | 95% CI | *P* | d | |
| Accept EMI component at t_n-1_ | -0.06 | | -0.12 - 0.00 | .06 | -0.06 | |
| Refuse EMI component at t_n-1_ | -0.06 | | -0.13 - 0.01 | .11 | -0.06 | |
| Negative affect at t_n-1_ | 0.41 | | 0.39 - 0.43 | <.001 | 0.46 | |
| Outcome: Stress at t_n_ | | | | | | |
|  | *b* | 95% CI | | *P* | | d |
| Accept EMI component at t_n-1_ | 0.02 | -0.04 - 0.08 | | .56 | | 0.02 |
| Refuse EMI component at t_n-1_ | 0.00 | -0.07 - 0.07 | | .99 | | 0.00 |
| Stress at t_n-1_ | 0.39 | 0.37 - 0.41 | | <.001 | | 0.43 |

^a^Adjusted for potential confounding by age, gender, allocation of EMI component, MRT, and psychological distress at baseline

**Table S2.** Sensitivity analysis for reason of EMI component non-initiation: Regression coefficients, 95% confidence intervals, *P*-values and effect sizes of linear mixed model for effects of actively accepting/ refusing a suggested EMI component vs no EMI component being suggested at t_n-1_ on well-being at t_n_ by well-being at tn-1 (hypothesis 2)^a^.

| Outcome: Positive affect at t_n_ | | | | |
| --- | --- | --- | --- | --- |
|  | *b* | 95% CI | *P* | *d* |
| Accept EMI component at t_n-1_ | 0.00 | -0.06 - 0.06 | .94 | 0.00 |
| Refuse EMI component at t_n-1_ | 0.01 | -0.06 - 0.08 | .69 | 0.02 |
| Positive affect at t_n-1_ | 0.38 | 0.33 - 0.44 | <.001 | 0.43 |
| Interaction Accept EMI component at t_n-1_ x Positive affect at t_n-1_ | 0.08 | 0.02 - 0.14 | .01 | 0.09 |
| High positive affect | 0.08 | -0.02 – 0.18 | .12 | 0.09 |
| Low positive affect | -0.08 | -0.18 – 0.02 | .15 | -0.9 |
| High vs low positive affect | 0.16 | 0.04 – 0.28 | .01 | 0.18 |
| Interaction Refuse EMI component at t_n-1_ x Positive affect at t_n-1_ | 0.07 | 0.00 - 0.13 | .05 | 0.08 |
| High positive affect | 0.08 | -0.03 – 0.19 | .21 | 0.09 |
| Low positive affect | -0.05 | -0.16 – 0.06 | .51 | -0.06 |
| High vs low positive affect | 0.13 | 0.00 – 0.26 | .05 | 0.15 |
| Outcome: Negative affect at t_n_ | | | | |
|  | *b* | 95% CI | *P* | *d* |
| Accept EMI component at t_n-1_ | -0.06 | -0.12 - 0.00 | .06 | -0.06 |
| Refuse EMI component at t_n-1_ | -0.06 | -0.13 - 0.01 | .10 | -0.07 |
| Negative affect at t_n-1_ | 0.48 | 0.43 - 0.54 | <.001 | 0.55 |
| Interaction Accept EMI component at t_n-1_ x Negative affect at t_n-1_ | -0.09 | -0.15 - -0.03 | <.01 | -0.10 |
| High negative affect | -0.14 | -0.24 - -0.03 | .01 | -0.15 |
| Low negative affect | 0.02 | -0.08 – 0.13 | .87 | 0.03 |
| High vs low negative affect | -0.16 | -0.29 - -0.02 | .02 | -0.18 |
| Interaction Refuse EMI component at t_n-1_ x Negative affect at t_n-1_ | -0.08 | -0.15 – 0.01 | .01 | -0.09 |
| High negative affect | -0.12 | -0.23 - -0.00 | .04 | -0.13 |
| Low negative affect | 0.02 | -0.09 – 0.14 | .89 | 0.03 |
| High vs low negative affect | -0.14 | -0.28 – 0.00 | .04 | -0.16 |
| Outcome: Stress at t_n_ | | | | |
|  | *b* | 95% CI | *P* | *d* |
| Accept EMI component at t_n-1_ | 0.02 | -0.04 - 0.08 | .59 | 0.02 |
| Refuse EMI component at t_n-1_ | 0 | -0.07 - 0.07 | .99 | 0.00 |
| Stress at t_n-1_ | 0.38 | 0.33 - 0.43 | <.001 | 0.42 |
| Interaction Accept EMI component at t_n-1_ x Negative affect at t_n-1_ | 0.03 | -0.03 - 0.09 | .32 | 0.03 |
| Interaction Refuse EMI component at t_n-1_ x Negative affect at t_n-1_ | -0.03 | -0.09 - 0.04 | .46 | -0.03 |

^a^Adjusted for potential confounding by age, gender, allocation of EMI component, MRT, and psychological distress at baseline

**Table S3.** Sensitivity analysis for reason of EMI component non-initiation: . Regression coefficients, 95% confidence intervals, *P*-values and effect sizes of linear mixed model for effects of accepting/ refusing a suggested EMI component at t_n-1_ vs no EMI component being suggested at t_n-1_ on change in resilience (mediator model, hypothesis 3)^a^.

| Outcome: Change in resilience | | | | |
| --- | --- | --- | --- | --- |
|  | *b* | 95% CI | *P* | *d* |
| Accept EMI component at t_n-1_ |  |  |  |  |
| Controlled for positive affect at tn-1 | -0.02 | -0.10 - 0.05 | .56 | -0.02 |
| Controlled for negative affect at tn-1 | -0.03 | -0.10 - 0.05 | .48 | -0.02 |
| Controlled for stress affect at tn-1 | -0.02 | -0.10 - 0.05 | .57 | -0.02 |
| Refuse EMI component at t_n-1_ |  |  |  |  |
| Controlled for positive affect at tn-1 | -0.06 | -0.15 - 0.03 | .19 | -0.05 |
| Controlled for negative affect at tn-1 | -0.06 | -0.15 - 0.03 | .20 | -0.05 |
| Controlled for stress affect at tn-1 | -0.06 | -0.15 - 0.03 | .22 | -0.05 |

^a^Adjusted for potential confounding by age, gender, allocation of EMI component, MRT, and psychological distress at baseline

**Table S4.** Sensitivity analysis for reason of EMI component non-initiation: . Regression coefficients, 95% confidence intervals, *P*-values and effect sizes of linear mixed model for effects of actively accepting/ refusing a suggested EMI component vs no EMI component being suggested at t_n-1_ on changes in well-being from t_n-1_ to t_n_ via changes in resilience from t_n-1_ to t_n_ (outcome model, hypothesis 3)^a^.

| Outcome: Change in positive affect | | | | |
| --- | --- | --- | --- | --- |
|  | *b* | 95% CI | *P* | *d* |
| Change in resilience | 0.23 | 0.21 - 0.24 | <.001 | 0.27 |
| Accept EMI component at t_n-1_ | 0.01 | -0.10 - 0.05 | .82 | 0.01 |
| Refuse EMI component at t_n-1_ | 0.03 | -0.15 - 0.03 | .43 | 0.03 |
| Outcome: Change in negative affect | | | | |
|  | *b* | 95% CI | *P* | *d* |
| Change in resilience | -0.16 | -0.18 - -0.15 | <.001 | -0.19 |
| Accept EMI component at t_n-1_ | -0.06 | -0.10 - 0.05 | .04 | -0.07 |
| Refuse EMI component at t_n-1_ | -0.07 | -0.15 - 0.03 | .06 | -0.08 |
| Outcome: Change in stress | | | | |
|  | *b* | 95% CI | *P* | *d* |
| Change in resilience | -0.11 | -0.17 - -0.13 | <.001 | -0.13 |
| Accept EMI component at t_n-1_ | 0.02 | -0.10 - 0.05 | .60 | 0.02 |
| Refuse EMI component at t_n-1_ | 0.00 | -0.15 - 0.03 | .93 | 0.00 |

^a^Adjusted for potential confounding by age, gender, allocation of EMI component, MRT, and psychological distress at baseline

**Table S5.** Sensitivity analysis for reason of EMI component non-initiation: Regression coefficients, 95% confidence intervals, and *P*-values of mediation model for actively accepting/ refusing a suggested EMI component vs no EMI component being suggested at t_n-1_ on changes in well-being from t_n-1_ to t_n_ via changes in resilience from t_n-1_ to t_n_ ( hypothesis 3)^a^.

| Outcome: Change in positive affect | | | |
| --- | --- | --- | --- |
|  | *b* | 95% CI | *P* |
| No EMI component vs Accept EMI component at t_n-1_ |  |  |  |
| Total effect | 0.00 | -0.06 - 0.06 | .99 |
| Direct effect | 0.01 | -0.05 - 0.06 | .84 |
| Indirect effect | 0.00 | -0.02 - 0.01 | .58 |
| Proportion mediated | 0.09 | -3.42 – 3.91 | .80 |
| No EMI component vs Refuse EMI component at t_n-1_ |  |  |  |
| Total effect | 0.05 | -0.03 - 0.12 | .24 |
| Direct effect | 0.07 | -0.01 - 0.14 | .07 |
| Indirect effect | -0.02 | -0.05 – 0.00 | .10 |
| Proportion mediated | -0.28 | -5.06 - 4.04 | .34 |
| Outcome: Change in negative affect | | | |
|  | *b* | 95% CI | *P* |
| No EMI component vs Accept EMI component at t_n-1_ |  |  |  |
| Total effect | -0.05 | -0.11 - 0.01 | .08 |
| Direct effect | -0.06 | -0.12 - 0.00 | .06 |
| Indirect effect | 0.00 | -0.01 - 0.02 | .52 |
| Proportion mediated | -0.07 | -0.96 – 0.44 | .59 |
| No EMI component vs Refuse EMI component at t_n-1_ |  |  |  |
| Total effect | -0.09 | -0.17 - -0.01 | .02 |
| Direct effect | -0.10 | -0.18 - -0.03 | <.01 |
| Indirect effect | 0.01 | 0.00 - 0.03 | .10 |
| Proportion mediated | -0.16 | -1.27 - 0.05 | .12 |
| Outcome: Change in stress | | | |
|  | *b* | 95% CI | *P* |
| No EMI component vs Accept EMI component at t_n-1_ |  |  |  |
| Total effect | 0.02 | -0.04 - 0.08 | .48 |
| Direct effect | 0.02 | -0.05 - 0.08 | .53 |
| Indirect effect | 0.00 | -0.01 - 0.01 | .65 |
| Proportion mediated | 0.06 | -1.27 - 1.81 | .74 |
| No EMI component vs Refuse EMI component at t_n-1_ |  |  |  |
| Total effect | 0.04 | -0.04 - 0.12 | .40 |
| Direct effect | 0.03 | -0.05 - 0.11 | .50 |
| Indirect effect | 0.01 | 0.00 - 0.02 | .13 |
| Proportion mediated | 0.11 | -1.76 - 1.37 | .46 |

^a^Adjusted for potential confounding by age, gender, allocation of EMI component, MRT, and psychological distress at baseline.
