# Supplementary material for: Promoting Psychological Resilience and Well-Being in Youth With a Smartphone-Based Ecological Momentary mHealth Intervention: Secondary Analysis of a Microrandomized Trial
Source: J Med Internet Res. 2026 Jun 18;28:e85552. doi: 10.2196/85552 (PMC13280375; doi:10.2196/85552)
Supplement: Multimedia Appendix 9 [file jmir-v28-e85552-s009.docx]

**Table S1.** Regression coefficients, 95% confidence intervals, *P*-values and effect sizes of linear mixed model for effect of initiation of an EMI component at t_n-1_ on change in resilience from t_n-1_ to t_n_: mediator model (hypothesis 3)^a^.

| Outcome: Change in resilience | | | | |
| --- | --- | --- | --- | --- |
|  | *b* | 95% CI | *p* | *d* |
| Initiation of EMI component at t_n-1_ |  |  |  |  |
| Controlled for positive affect at t_n-1_ | 0.02 | -0.03 - 0.07 | .36 | 0.02 |
| Controlled for negative affect at t_n-1_ | 0.02 | -0.03 - 0.06 | .52 | 0.01 |
| Controlled for stress affect at t_n-1_ | 0.02 | -0.03 - 0.07 | .36 | 0.02 |

^a^Adjusted for potential confounding by age, gender, allocation of EMI component, MRT, and psychological distress at baseline.

**Table S2.** Regression coefficients, 95% confidence intervals, *P*-values and effect sizes of linear mixed model for effects of initiating an EMI component at t_n-1_ on changes in well-being from at t_n-1_ to at t_n_  via changes in resilience from at t_n-1_ to at t_n_ : outcome model (hypothesis 3)^a^.

| Outcome: Change in positive affect | | | | |
| --- | --- | --- | --- | --- |
|  | *b* | 95% CI | *P* | *d* |
| Change in resilience | 0.22 | 0.21 - 0.24 | <.001 | 0.27 |
| Initiation of EMI component at t_n-1_ | -0.00 | -0.04 - 0.03 | .86 | 0.00 |
| Outcome: Change in negative affect | | | | |
|  | *b* | 95% CI | *P* | *d* |
| Change in resilience | -0.16 | -0.18 - -0.15 | <.001 | -0.19 |
| Initiation of EMI component at t_n-1_ | -0.02 | -0.06 - 0.01 | .19 | -0.03 |
| Outcome: Change in stress | | | | |
|  | *b* | 95% CI | *P* | *d* |
| Change in resilience | -0.15 | -0.17 - -0.13 | <.001 | -0.14 |
| Initiation of EMI component at t_n-1_ | 0.03 | -0.01 - 0.08 | .15 | 0.03 |

^a^Adjusted for potential confounding by age, gender, allocation of EMI component, MRT, and psychological distress at baseline.
